# Emergency minimally invasive versus open left-sided colonic resection for complicated diverticulitis: a systematic review and meta-analysis

**DOI:** 10.1186/s12893-026-04043-3

**Published:** 2026-07-21

**Authors:** Hariruk Yodying, Vichit Viriyaroj, Thammanij Rookkachart, Thana Boonsinsukh, Anuwat Chartkitcharoen, Wannakorn Prapasajchavet, Natchanok Mekrugsakit, Patcharaon Petchkaewkul, Ratchanon Laojanun

**Affiliations:** Department of Surgery, HRH Princess Maha Chakri Sirindhorn Medical Center, Faculty of Medicine, Srinakharinwirot University, Nakhon Nayok, Thailand

**Keywords:** Diverticulitis, Emergency surgery, Laparoscopic surgery, Minimally invasive surgery, Meta-analysis, Systematic review

## Abstract

**Background:**

Emergency surgery for complicated diverticulitis carries substantial morbidity and mortality. Whether minimally invasive approaches offer advantages over open surgery in this urgent setting remains uncertain. We synthesized contemporary evidence comparing emergency minimally invasive versus open left-sided colonic resection for complicated diverticulitis.

**Methods:**

Following PRISMA 2020 guidelines and a prospectively registered protocol, we searched PubMed, Scopus, Cochrane Library, and ClinicalTrials.gov through July 2025. We included comparative studies of emergency minimally invasive versus open colectomy for complicated diverticulitis (Hinchey II–IV). Random-effects meta-analysis with Hartung-Knapp-Sidik-Jonkman adjustment was performed. Risk of bias was assessed using ROBINS-I at the outcome level, and certainty of evidence was evaluated using GRADE.

**Results:**

Six studies encompassing 9,637 patients (1,821 minimally invasive, 7,816 open) met inclusion criteria. Minimally invasive surgery was associated with lower 30-day mortality (4.5% vs. 8.7%; RR 0.48, 95% CI 0.40–0.59; *P* = 0.0002; I²=0%; low certainty); this estimate derived 86.9% of its weight from one database study and was no longer statistically significant when that study was excluded (RR 0.61, 95% CI 0.35–1.06). Major complications did not differ significantly (15.2% vs. 17.7%; RR 0.63, 95% CI 0.36–1.08; *P* = 0.10; very low certainty). Length of stay was shorter (MD − 2.55 days, 95% CI − 4.37 to − 0.74; *P* = 0.02) and incisional surgical site infection rates were lower (RR 0.30, 95% CI 0.12–0.75; *P* = 0.02), though operative time was longer (MD + 18.27 min, 95% CI 0.89–35.64; *P* = 0.04). Reoperation rates were similar (RR 0.98, 95% CI 0.78–1.22).

**Conclusions:**

Emergency minimally invasive resection was associated with lower 30-day mortality and shorter hospital stay, without conclusive evidence of fewer major complications. The mortality estimate derived most of its statistical weight from one large database study; after excluding that study, the point estimate remained favorable but imprecise. Overall certainty was low to very low, so these findings are hypothesis-generating rather than practice-changing. Minimally invasive resection may be considered in hemodynamically stable, selected patients operated on by surgeons with advanced laparoscopic colorectal expertise, pending prospective validation.

**Systematic review registration:**

PROSPERO CRD420251142546.

**Supplementary Information:**

The online version contains supplementary material available at https://doi.org/10.1186/s12893-026-04043-3.

## Introduction

Diverticular disease represents a major global health burden, with the highest incidence in Western industrialized nations [1]. In the United States alone, diverticular disease generates over 450,000 emergency department visits and 200,000 hospital admissions annually, while European estimates indicate approximately 23,000 associated deaths per year [2–4]. The incidence has increased particularly among younger populations over recent decades, with complicated presentations requiring emergency intervention becoming increasingly common worldwide [5, 6]. When diverticulitis becomes complicated by perforation, abscess, or peritonitis, emergency surgical intervention frequently carries mortality rates ranging from 5% to 20% depending on disease severity and patient factors [7, 8]. 

The role of minimally invasive surgery in elective colorectal procedures is well established. Multiple randomized trials and systematic reviews have demonstrated equivalent oncologic outcomes alongside reduced wound complications, shorter hospital stays, and faster recovery compared with open surgery [9–11]. Enhanced recovery protocols further amplify these benefits when combined with laparoscopic approaches [12]. However, extrapolating these benefits to the emergency setting has been controversial. Concerns regarding technical difficulty in the presence of inflammation, the need for rapid operative intervention, and patient hemodynamic instability have historically limited adoption of laparoscopic approaches for acute complicated diverticulitis [13, 14]. 

Recent advances in laparoscopic technique, improved perioperative care protocols, and accumulating institutional experience have gradually shifted practice patterns [15, 16]. The 2020 World Society of Emergency Surgery guidelines acknowledge that laparoscopic sigmoid resection may be performed safely in emergency settings by experienced surgeons, though recommendations remain weak due to limited comparative evidence [7]. The European Association for Endoscopic Surgery and the American Society of Colon and Rectal Surgeons have issued similar cautious endorsements [17, 18]. Most recently, the 2025 EAES rapid guideline with ESCP participation provided updated recommendations supporting minimally invasive approaches in selected emergency cases [19]. 

Several large database studies and institutional series comparing minimally invasive and open approaches for emergency diverticulitis have emerged [20–25]. Prior systematic reviews have examined laparoscopic versus open colorectal surgery in emergency settings more broadly, but these syntheses included heterogeneous indications or substantial proportions of elective cases [26–28]. No recent synthesis restricted to contemporary emergency left-sided resection for Hinchey II to IV diverticulitis has specifically addressed this question. Our review differs from these prior syntheses in several respects. Cirocchi et al., [26] pooled approximately 436 patients from a smaller and partly older study set; Wu et al., [27] combined elective and emergency cohorts; and Warps et al., [28] examined emergency laparoscopic colorectal surgery across heterogeneous indications rather than diverticulitis specifically. We restricted eligibility to emergency left-sided resection for Hinchey II to IV disease, excluded studies of laparoscopic lavage without resection, and applied a pre-specified algorithm to handle overlapping national-database populations, retaining only the largest and most rigorously adjusted dataset and excluding five temporally overlapping NSQIP studies.

We therefore conducted a systematic review and meta-analysis to evaluate whether emergency minimally invasive left-sided colonic resection for complicated diverticulitis is associated with improved outcomes compared with open surgery. Our analysis focused on mortality, major complications, and clinically meaningful secondary endpoints while carefully addressing the methodological challenges inherent to synthesizing observational surgical evidence. The clinical question is not whether minimally invasive surgery is superior to open surgery in general, but whether an intention to attempt minimally invasive resection, in hemodynamically stable and appropriately selected patients at centers with established laparoscopic colorectal expertise, is associated with improved outcomes compared with upfront open surgery. This distinction matters because allocation to surgical approach in all available studies is non-random and strongly influenced by patient selection, so any observed difference reflects both the approach and the characteristics of the patients chosen for it.

## Methods

### Protocol and registration

This systematic review and meta-analysis adhered to Preferred Reporting Items for Systematic Reviews and Meta-Analyses (PRISMA) 2020 guidelines [29]. The protocol was prospectively registered with PROSPERO (CRD420251142546) prior to data extraction and is available at https://www.crd.york.ac.uk/prospero/. Detailed search strategies, excluded studies with reasons, risk of bias assessments, and additional statistical analyses are provided in the Supplementary Materials.

### Eligibility criteria

We included comparative studies of adults (≥ 18 years) undergoing emergency left-sided colonic resection for complicated diverticulitis (Hinchey classification II–IV) that compared minimally invasive surgery (laparoscopic, hand-assisted, or robotic) with open surgery. Emergency surgery was defined as unplanned operation during the index hospitalization for acute complicated diverticulitis, encompassing both immediate surgery (within 24 h) and urgent surgery after initial resuscitation or failed non-operative management (Supplementary Text S1) [30]. Studies reporting at least one primary outcome (30-day mortality or major complications) were eligible. We excluded case reports, series without a comparator group, studies combining elective and emergency cases without separate outcome reporting, and studies focusing exclusively on laparoscopic lavage without resection.

### Information sources and search strategy

We systematically searched PubMed, Scopus, Cochrane Central Register of Controlled Trials, and ClinicalTrials.gov from database inception through July 31, 2025. The search strategy combined controlled vocabulary (MeSH terms) and keywords for three concept domains: diverticulitis/diverticular disease, emergency/urgent surgery, and minimally invasive/laparoscopic surgery (Supplementary Table S1). Reference lists of included studies and relevant systematic reviews were manually screened to identify additional eligible studies. No language restrictions were applied.

### Study selection and data extraction

Two reviewers independently screened titles and abstracts using Rayyan systematic review software [31], followed by full-text review of potentially eligible studies. Disagreements were resolved through discussion. Inter-rater reliability was excellent (κ = 0.92 for title/abstract screening, κ = 0.88 for full-text assessment; Supplementary Table S7) [32]. 

For studies using overlapping populations from the same database, we applied a pre-specified algorithm retaining only the largest, most recent study with the most rigorous statistical adjustment [33]. This resulted in exclusion of five NSQIP-based studies with temporal overlap with Le et al., [20] which represented the largest and most rigorously adjusted analysis of that database (Supplementary Table S2).

Data extraction was performed independently by two reviewers using a standardized form. Extracted variables included study characteristics, patient demographics, disease severity indicators (Hinchey classification), surgical details (procedure type, conversion rates), and all reported outcomes. Discrepancies were reconciled by returning to source documents.

### Outcomes

The primary outcomes were 30-day mortality (or in-hospital mortality when 30-day data were unavailable) and major postoperative complications (Clavien-Dindo grade III or higher, excluding mortality) [34]. For studies not explicitly reporting 30-day mortality, in-hospital mortality was accepted as a surrogate (Cassini et al., [24] Paasch et al., [25]; Supplementary Table S5). Major complications were harmonized to Clavien-Dindo grade III–IV and overall morbidity to grade I–IV, excluding death; the NSQIP composite “major adverse events” from Le et al., [20] was not pooled for these outcomes due to non-comparable definitions (Supplementary Table S5). Secondary outcomes included overall morbidity (Clavien-Dindo I–IV), unplanned reoperation, length of hospital stay, operative time, surgical site infections (incisional and organ-space), stoma formation, and anastomotic leak.

### Risk of bias assessment

Risk of bias was assessed at the outcome level using the Risk of Bias in Non-randomized Studies of Interventions (ROBINS-I) tool [35]. This approach recognizes that different outcomes within the same study may be susceptible to different forms of bias. For each study-outcome combination, we evaluated seven domains: confounding, selection of participants, classification of interventions, deviations from intended interventions, missing data, measurement of outcomes, and selection of reported results. Given the observational nature of all included studies, we conservatively rated confounding bias as at least “moderate” for all outcomes, acknowledging that residual confounding by indication is inherent to this study design. Detailed domain-level assessments with rationale are provided in Supplementary Table S3.

### Statistical analysis

Meta-analyses were performed using random-effects models with the Hartung-Knapp-Sidik-Jonkman (HKSJ) adjustment for confidence intervals, which uses a t-distribution and generally provides more appropriate coverage than the standard DerSimonian-Laird approach when few studies are pooled [36, 37]. Because the HKSJ method can itself become anti-conservative when the number of studies is small and between-study heterogeneity is estimated as zero, producing an interval narrower than the conventional interval, for such an outcome (major complications, three studies with I²=0%) we instead reported the conventional DerSimonian-Laird (Wald-type) random-effects confidence interval [37]. For dichotomous outcomes, we calculated risk ratios using the Mantel-Haenszel method with DerSimonian-Laird between-study variance estimation. For continuous outcomes, we calculated mean differences using inverse-variance weighting. When studies reported medians with interquartile ranges, we estimated means and standard deviations using validated conversion methods (Supplementary Table S6) [38, 39]. For studies with zero events in one arm, we applied a 0.5 continuity correction; studies with zero events in both arms were excluded from that specific outcome analysis (Supplementary Text S2) [40]. Outcomes were analyzed using each source study’s reported arm definitions. Most studies retained converted cases within the minimally invasive arm, but conversion handling was not uniform (for example, one study reporting a totally laparoscopic Hartmann procedure recorded conversions within the open arm), and it could not be fully standardized; this is addressed as a limitation.

Heterogeneity was assessed using the I² statistic and Cochran’s Q test [41]. We considered I² values of 0–40% as potentially unimportant, 30–60% as moderate, 50–90% as substantial, and 75–100% as considerable heterogeneity [42]. Pre-specified sensitivity analyses included exclusion of the largest study to assess influence, restriction to propensity score-matched studies, restriction to studies from 2015 or later, and analysis by disease severity (Supplementary Table S4). Publication bias was assessed using funnel plots, Egger’s regression test, and Begg’s rank correlation test for outcomes with at least 6 studies [43, 44]. 

To address the possibility that our pooled estimates were driven by a single large study, we performed leave-one-out analyses for mortality, presenting results both with and without the dominant study. We did not calculate number needed to treat (NNT) because this metric implies a causal interpretation that cannot be justified from observational data where residual confounding is unavoidable [45]. 

### Certainty of evidence

Certainty of evidence was assessed using the Grading of Recommendations Assessment, Development and Evaluation (GRADE) framework [46]. For each outcome, we started at “low” certainty (reflecting the observational study designs) and considered downgrading for risk of bias, inconsistency, indirectness, imprecision, and publication bias. We considered upgrading for large magnitude of effect, dose-response gradients, or when plausible confounding would reduce the demonstrated effect [47]. GRADE assessments were performed using GRADEpro Guideline Development Tool software.

## Results

### Study selection

**Fig. 1:**
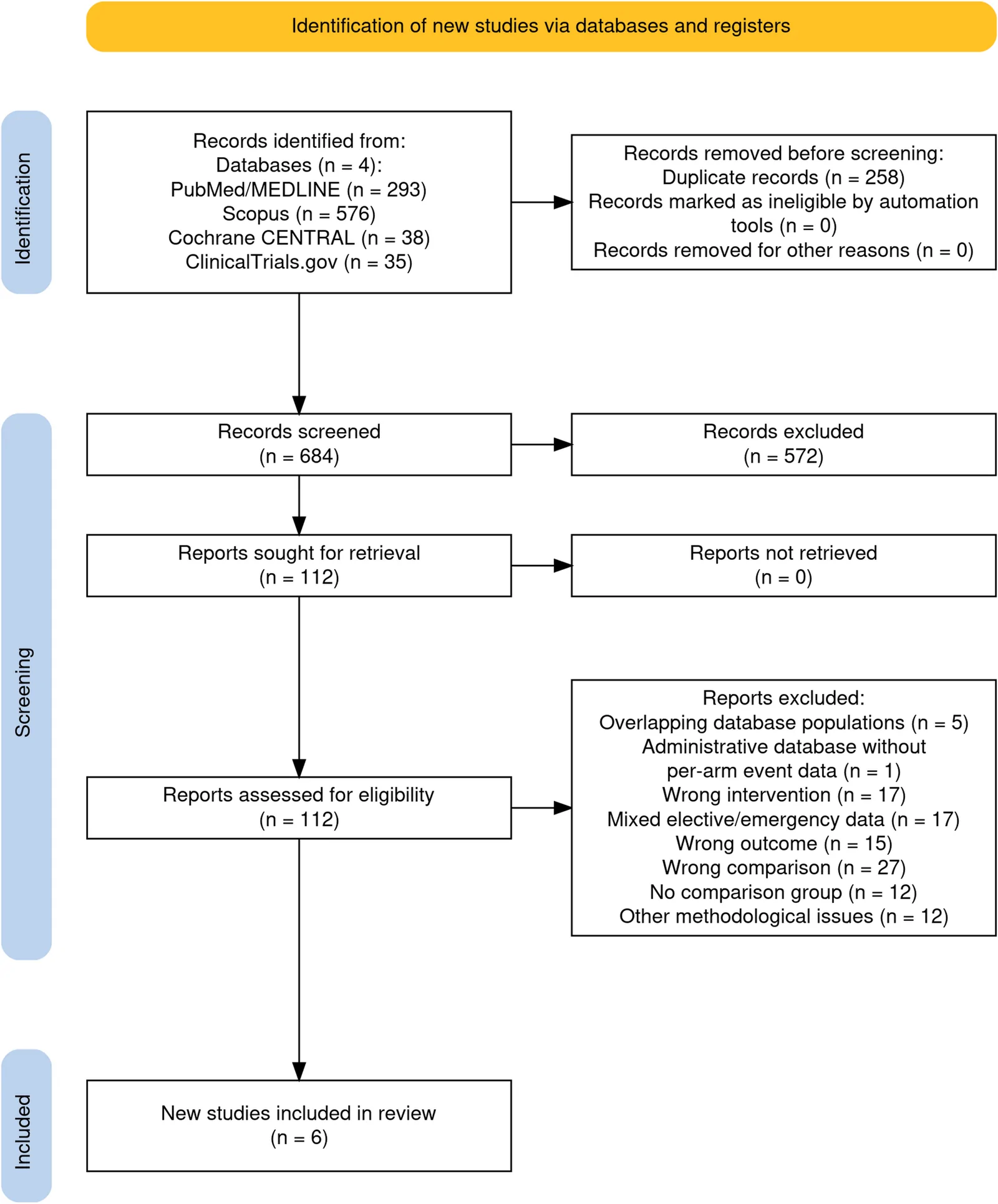
PRISMA 2020 flow diagram. Systematic study selection process from 942 database records to 6 included studies (9,637 patients). Five NSQIP studies (Amodu 2024, Moghadamyeghaneh 2021, Lee 2020, Monzavi 2021, Hajirawala 2022) excluded due to temporal overlap with Le 2024 (2015–2020), retained as largest with most rigorous adjustment. Rea 2012 (*n*=67,645) excluded for incompatible data structure (administrative national estimates without extractable per-arm event data); earlier era and an approximately 55% conversion rate are supporting context. MIS: minimally invasive surgery

Our systematic search identified 942 citations across four databases. After removing 258 duplicates, we screened 684 unique records by title and abstract, excluding 572 records primarily for wrong population, case series without comparison groups, or purely elective surgery. Full-text assessment of 112 articles led to exclusion of 106 studies: five for overlapping NSQIP database populations, one study (Rea et al.,) that reported weighted national-estimate data with adjusted odds ratios rather than patient-level counts, precluding compatible pooling, and that reflected early-era practice with an approximately 55% conversion rate, 17 for lavage-only interventions without resection, 17 for inseparable mixed elective-emergency cohorts, 15 for missing relevant outcomes, 27 for wrong comparisons (Hartmann versus primary anastomosis rather than minimally invasive versus open approach), 12 for single-arm design, and 12 for other methodological limitations including unpublished conference abstracts. Detailed reasons for excluding all 106 full-text articles are documented in Supplementary Table S2. Six studies encompassing 9,637 patients met all eligibility criteria and were included in quantitative synthesis (Fig. 1) [20–25]. 

### Study characteristics

The six included studies were published between 2013 and 2024 and encompassed patients treated from 2000 to 2020 across North America and Europe (Table 1) [20–25]. Two studies used national database populations (Le et al., [20] *n* = 9,124; Turley et al., [21] *n* = 134), while four were institutional cohorts from the Netherlands [22], Canada [23], Italy [24], and Germany [25]. The minimally invasive group comprised 1,821 patients (18.9%) and the open surgery group 7,816 patients (81.1%).

**Table 1:** Characteristics of included studies

Study	Country	Period	Design	*N* (MIS: Open)	Hinchey	Adjustment Method	Conversion Rate
Le 2024 [20]	USA	2015–2020	Retrospective cohort	9,124 (1,580:7,544)	II–IV	Entropy balancing	33.7%
Turley 2013 [21]	USA	2005–2009	PSM cohort	134 (67:67)	Mixed	PSM 1:1	NR
Vennix 2016 [22]	Netherlands	2010–2014	PSM cohort	117 (39:78)	III–IV	PSM 1:2	4.5%
Letarte 2015 [23]	Canada	2000–2011	Retrospective cohort	125 (39:86)	II–IV	None	5.1%
Cassini 2017 [24]	Italy	2008–2016	Retrospective cohort	60 (36:24)	III–IV	None	10.0%
Paasch 2022 [25]	Germany	2007–2019	Retrospective cohort	77 (60:17)	III–IV	Multivariable	15.0%

*MIS* Minimally invasive surgery, *NR* Not reported, *PSM* Propensity score matching

All studies included patients with complicated diverticulitis requiring emergency surgery. Disease severity ranged from Hinchey II through IV across studies. Surgical approaches in the minimally invasive group included conventional laparoscopy and hand-assisted techniques; robotic-assisted procedures were included in Le et al., [20] Conversion rates ranged from 4.5% to 33.7% across studies, with the higher rates in contemporary database studies likely reflecting inclusive definitions and appropriate surgical judgment in challenging emergency cases (Supplementary Text S3). Three studies employed propensity score matching or entropy balancing to reduce confounding (Le et al., [20] Vennix et al., [22] Turley et al., [21]), while three used unadjusted comparisons or multivariable regression (Letarte et al., [23] Cassini et al., [24] Paasch et al., [25]).

Patient demographics and operative characteristics across studies are presented in Table 2. The Le et al., [20] cohort reflected mixed contemporary practice, with stomas created in 35.8% of minimally invasive versus 84.8% of open surgery patients. Operative times were consistently longer for minimally invasive approaches, with Le et al., [20] reporting medians of 158 min (119–210) versus 128 min (95–172), while Letarte et al. [23] documented means of 273.6 ± 60.3 min versus 241.8 ± 65.5 min.

**Table 2:** Patient demographics and operative characteristics

Variable	Le 2024 [20]	Vennix 2016 [22]	Turley 2013 [21]	Letarte 2015 [23]	Cassini 2017 [24]	Paasch 2022 [25]
Age, years						
MIS	45.5 [36–54]^a^	56.2 (14.2)^b^	58.5 (16.3)^b^	61.6 (13.7)^b^	66.5 (30–86)^c^	67 [50–77]^a^
Open	48.5 [39–58]^a^	56.4 (13.3)^b^	59.4 (13.5)^b^	60.9 (12.0)^b^	65.8 (41–96)^c^	73 [63–78]^a^
Male, %						
MIS	47.6	35.9	61.2	30.8	39	47
Open	47.1	30.8	59.7	61.6	25	47
BMI, kg/m²						
MIS	78.7% ≥25^d^	25.3 (3.5)^b^	27.6 (7.8)^b^	26.3 (4.0)^b^	NR	27.0 [24.3–29.5]^a^
Open	75.3% ≥25^d^	27.1 (5.8)^b^	26.7 (6.5)^b^	26.7 (4.5)^b^	NR	25.3 [23.5–34.9]^a^
ASA ≥ III, %						
MIS	67.0	38.7	52.2	20.5	100	50.0
Open	78.4	23.6	59.7	24.4	100	88.2
Hinchey III/IV, %	Not staged	79.5/20.5	Not staged	Mixed	43.3/56.7	~ 100^e^
Operative time, min						
MIS	158 [119–210]^a^	127 (105–159)^a^	NR	273.6 (60.3)^b^	170 (28)^b^	120 [90–162]^a^
Open	128 [95–172]^a^	96.5 (87–120)^a^	NR	241.8 (65.5)^b^	170 (35)^b^	100 [69–156]^a^
Hartmann, %						
MIS	35.8	66.7	100	5.1	100	63.3
Open	84.8	65.4	100	22.1	100	82.4

*ASA* American Society of Anesthesiologists, *BMI* Body mass index, *MIS * Minimally invasive surgery, *NR * Not reported

^a^Median [interquartile range]; ^b^Mean (standard deviation); ^c^Mean (range); ^d^Percentage ≥ 25 kg/m²; ^e^Perforated complicated diverticulitis (clinically equivalent to Hinchey III–IV)

### Risk of bias

Risk of bias assessments at the outcome level are presented in Supplementary Table S3. For the primary outcomes (mortality and major complications), studies using propensity score matching or entropy balancing (Le et al., [20] Vennix et al., [22] Turley et al., [21]) were rated as having moderate overall risk of bias, while unadjusted studies (Letarte et al., [23] Cassini et al., [24] Paasch et al., [25]) were rated as serious. Confounding was rated as moderate or serious across all studies, reflecting the inherent selection bias in observational comparisons of surgical approaches. Bias in outcome measurement was generally low for objective outcomes (mortality, reoperation) but moderate for outcomes requiring clinical judgment (complications grading).

### Primary outcomes

**Fig. 2:**
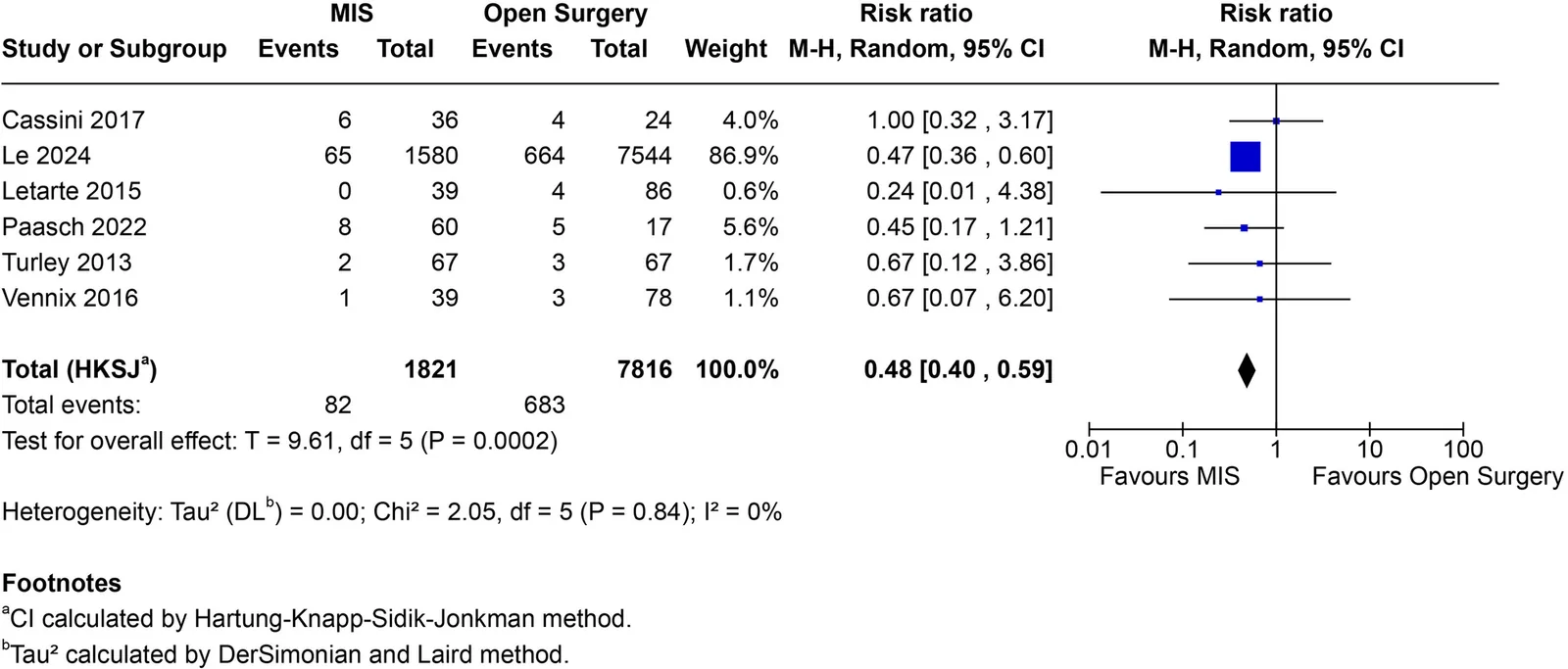
Forest plot − 30-day mortality. Forest plot of 30-day mortality comparing minimally invasive versus open surgery for emergency complicated diverticulitis. Six studies (9,637 patients) demonstrated RR 0.48 (95% CI 0.40–0.59; *P* = 0.0002; I²=0%). Le 2024 contributes 86.9% of the weight. Five of six studies favor minimally invasive surgery; Cassini 2017 shows equal mortality (RR 1.00). Sensitivity analysis excluding Le 2024 is presented in Supplementary Figure S1

30-Day Mortality. All six studies reported mortality, with timing specified as 30-day in four studies and in-hospital in two studies [20–25]. Minimally invasive surgery was associated with significantly lower mortality: 82 of 1,821 patients (4.5%) versus 683 of 7,816 (8.7%); risk ratio 0.48 (95% CI 0.40–0.59); *P* = 0.0002; I²=0% (Fig. 2). There was no statistical heterogeneity. Five of the six studies had point estimates favoring minimally invasive surgery, whereas Cassini et al., [24] reported equal mortality in both arms (16.6%; RR 1.00).

The Le et al., [20] study contributed 86.9% of the weight to this analysis owing to its large sample size (*n* = 9,124). Sensitivity analysis excluding this study yielded a directionally consistent but statistically non-significant result: RR 0.61 (95% CI 0.35–1.06); *P* = 0.07; I²=0% (Supplementary Figure S1). When restricted to the three studies employing advanced statistical adjustment (entropy balancing or propensity score matching: Le et al., [20] Vennix et al., [22] Turley et al., [21]), the association remained similar: RR 0.49 (95% CI 0.40–0.60); *n* = 9,375; I²=0% (Supplementary Table S4). Subgroup analysis of Hinchey III–IV patients (Vennix et al., [22] Cassini et al., [24] Paasch et al., [25]) showed RR 0.60 (95% CI 0.33–1.10). Leave-one-out analysis confirmed that Le et al., [20] was the only study whose exclusion substantially altered conclusions (Supplementary Table S4). In absolute terms, the lower mortality corresponded to a crude absolute difference of 4.2% points (4.5% versus 8.7%). Le 2024, a large NSQIP-based cohort with entropy balancing, contributed 86.9% of the weight for 30-day mortality and 36.7% for incisional SSI, making its methodological strengths and limitations critical for interpretation.

**Fig. 3:**
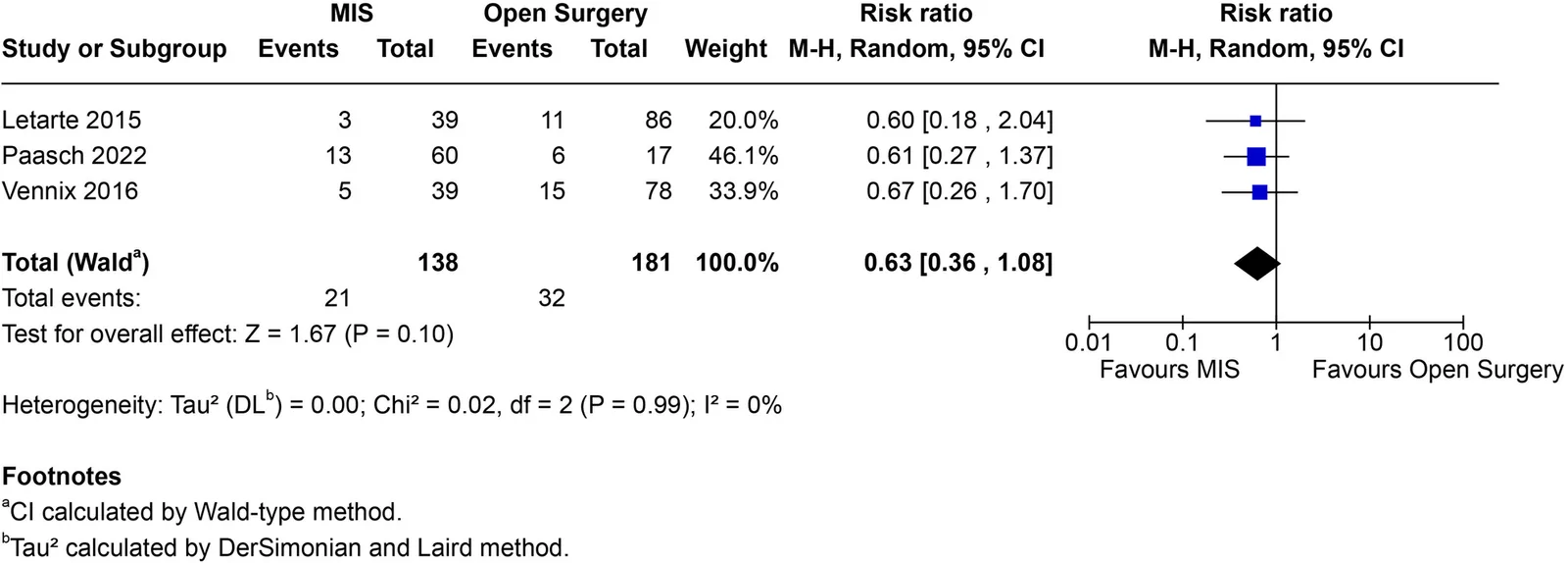
Forest plot - major complications (Clavien-Dindo ≥ III excluding mortality). Forest plot of major complications (Clavien-Dindo grade ≥III, excluding mortality) comparing minimally invasive versus open surgery. Three studies (319 patients). The unmodified HKSJ estimate (RR 0.63, 95% CI 0.55–0.72) was anti-conservative at three studies with I²=0%; the conventional DerSimonian-Laird (Wald-type) random-effects interval is reported (RR 0.63, 95% CI 0.36–1.08; P=0.10; I²=0%). Le 2024 was excluded due to incompatible outcome definition. Each contributing study individually crossed the null

Major Complications. Three studies reported major complications using Clavien-Dindo grade III or higher (excluding mortality) among 319 patients [22, 23, 25]. Le et al., [20] was excluded from this outcome because it reported a composite “major adverse events” measure incompatible with the Clavien-Dindo classification. Turley et al., [21] was excluded as it did not use Clavien-Dindo grading. Major complications occurred in 21 of 138 (15.2%) minimally invasive versus 32 of 181 (17.7%) open patients (Fig. 3). The pooled risk ratio was 0.63; however, with only three studies and zero estimated heterogeneity, the unmodified Hartung-Knapp-Sidik-Jonkman interval (0.55–0.72) was anti-conservative, being narrower than the conventional random-effects interval. The conventional DerSimonian-Laird (Wald-type) random-effects interval was therefore reported for this outcome, and the difference was not statistically significant (RR 0.63, 95% CI 0.36–1.08; *P* = 0.10; I²=0%). The observed absolute difference was modest (15.2% versus 17.7%); the study-stratified relative estimate is lower than this crude difference because one study (Paasch) contributed a reversed allocation ratio with high event rates, so the two figures should be read together.

### Secondary outcomes

**Fig. 4:**
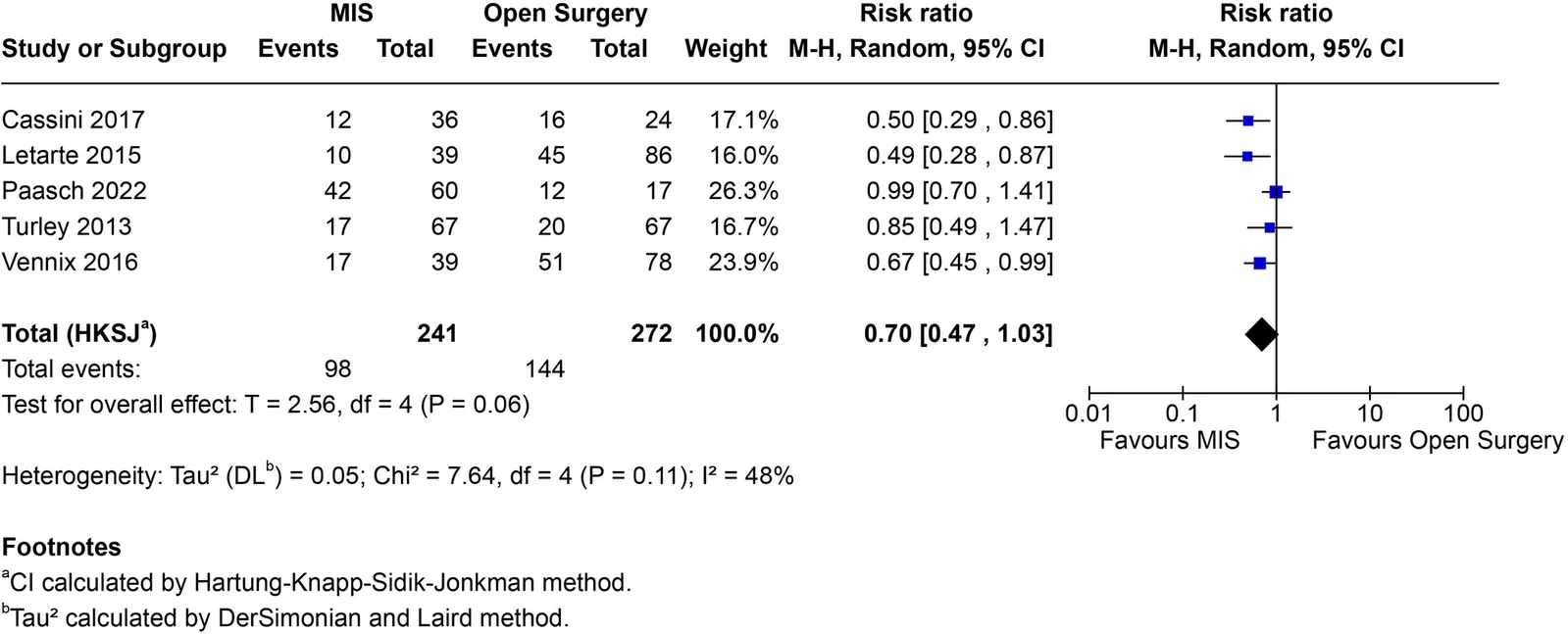
Forest plot - overall morbidity (Clavien-Dindo I-IV). Forest plot of overall morbidity (Clavien-Dindo grade I–IV, excluding mortality). Five studies (513 patients) showed no statistically significant difference, although the point estimate favored minimally invasive surgery: RR 0.70 (95% CI 0.47–1.03; P=0.06; I²=48%). Le 2024 was excluded due to non-comparable composite outcome definition. Moderate heterogeneity reflects varying outcome definitions and follow-up protocols across studies

Overall Morbidity. Five studies reported overall postoperative morbidity (Clavien-Dindo I–IV, excluding mortality) [21–25]. Le et al., [20] was excluded due to a non-comparable composite definition. No statistically significant difference was observed, although the point estimate favored minimally invasive surgery: 98 of 241 (40.7%) versus 144 of 272 (52.9%); RR 0.70 (95% CI 0.47–1.03); *P* = 0.06; I²=48%; low-certainty evidence (Fig. 4).

**Fig. 5:**
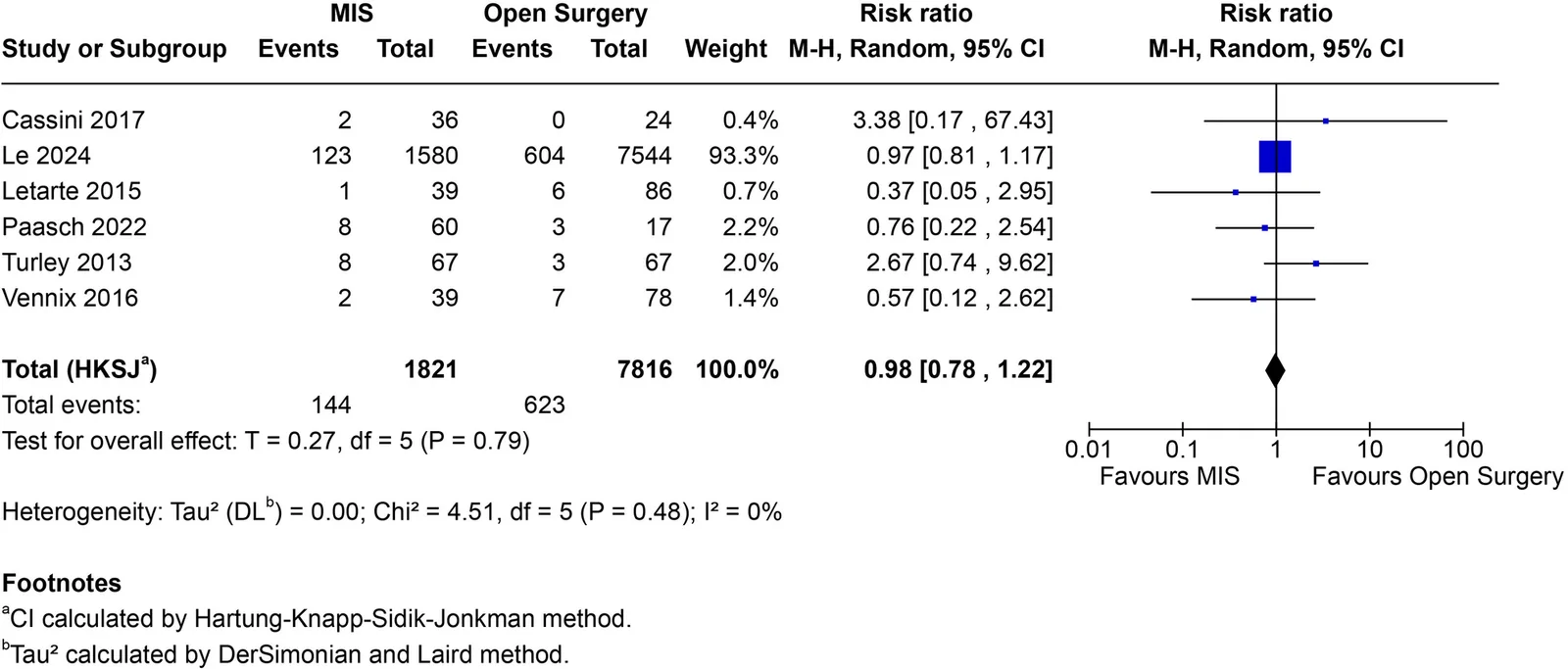
Forest plot - unplanned reoperation. Forest plot of unplanned reoperation during index hospitalization. Six studies (9,637 patients) demonstrated no significant difference between groups: RR 0.98 (95% CI 0.78–1.22; P=0.79; I²=0%). Reoperation indications distributed similarly across both groups, including bleeding control, anastomotic complications, and wound dehiscence

Reoperation. All six studies reported unplanned reoperation rates during the index hospitalization [20–25]. No significant difference was observed: 144 of 1,821 (7.9%) versus 623 of 7,816 (8.0%); RR 0.98 (95% CI 0.78–1.22); *P* = 0.79; I²=0% (Fig. 5).

**Fig. 6:**
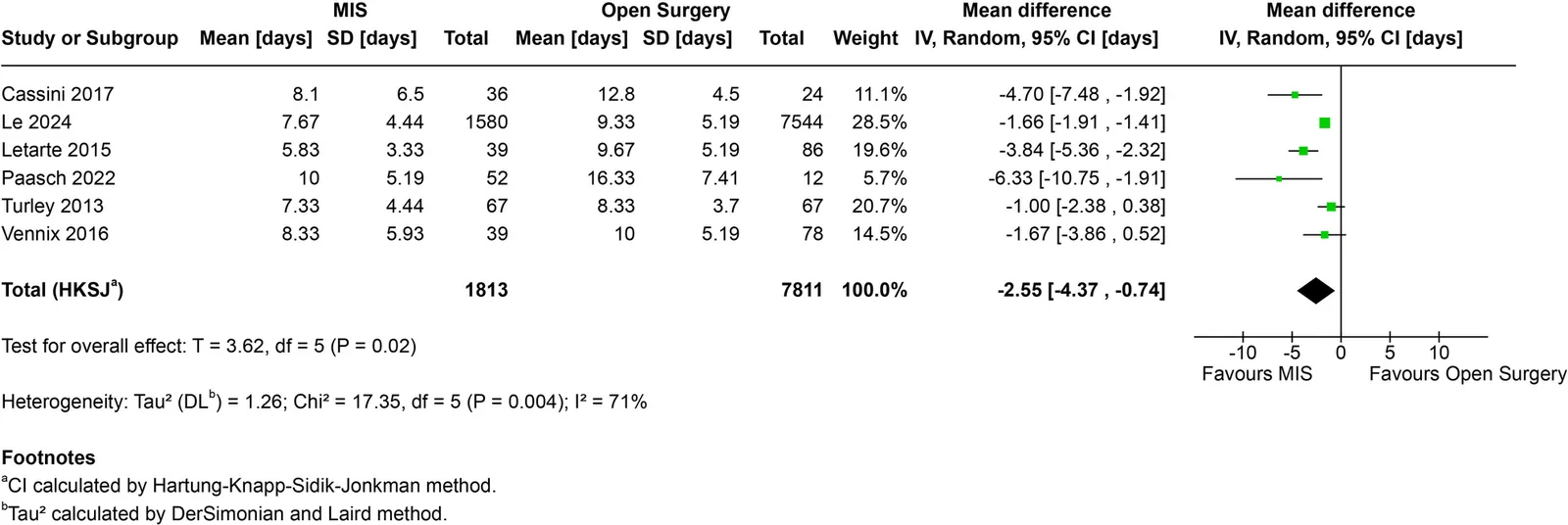
Forest plot - length of hospital stay. Forest plot of length of hospital stay (days). Six studies showed minimally invasive surgery was associated with shorter hospitalization: mean difference −2.55 days (95% CI −4.37 to −0.74; P=0.02; I²=71%). Substantial heterogeneity reflects variation in discharge protocols across healthcare systems and geographic regions

Length of Hospital Stay. Minimally invasive surgery was associated with significantly shorter hospitalization: mean difference − 2.55 days (95% CI − 4.37 to − 0.74); *P* = 0.02; I²=71% (Fig. 6). The substantial heterogeneity reflected variations in discharge protocols and healthcare systems across studies.

**Fig. 7:**
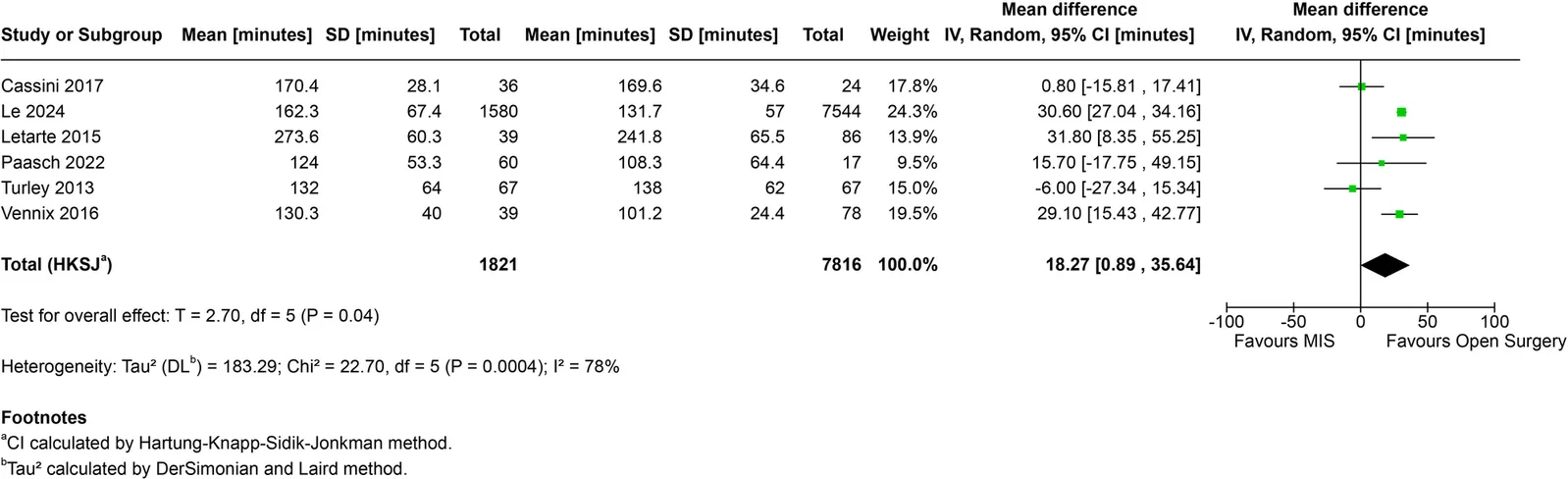
Forest plot - operative time. Forest plot of operative time (minutes). Across six studies, minimally invasive surgery was associated with longer operative time: mean difference +18.27 minutes (95% CI 0.89–35.64; P=0.04; I²=78%), using Turley's propensity-matched cohort. Substantial heterogeneity reflects variation in surgical techniques and case complexity

Operative Time. Minimally invasive surgery was associated with longer operative time: mean difference + 18.27 min (95% CI 0.89–35.64); *P* = 0.04; I²=78% (Fig. 7), using Turley’s propensity-matched cohort. Excluding Turley, the estimate was similar (mean difference + 23.1 min, 95% CI 6.4–39.7).

**Fig. 8:**
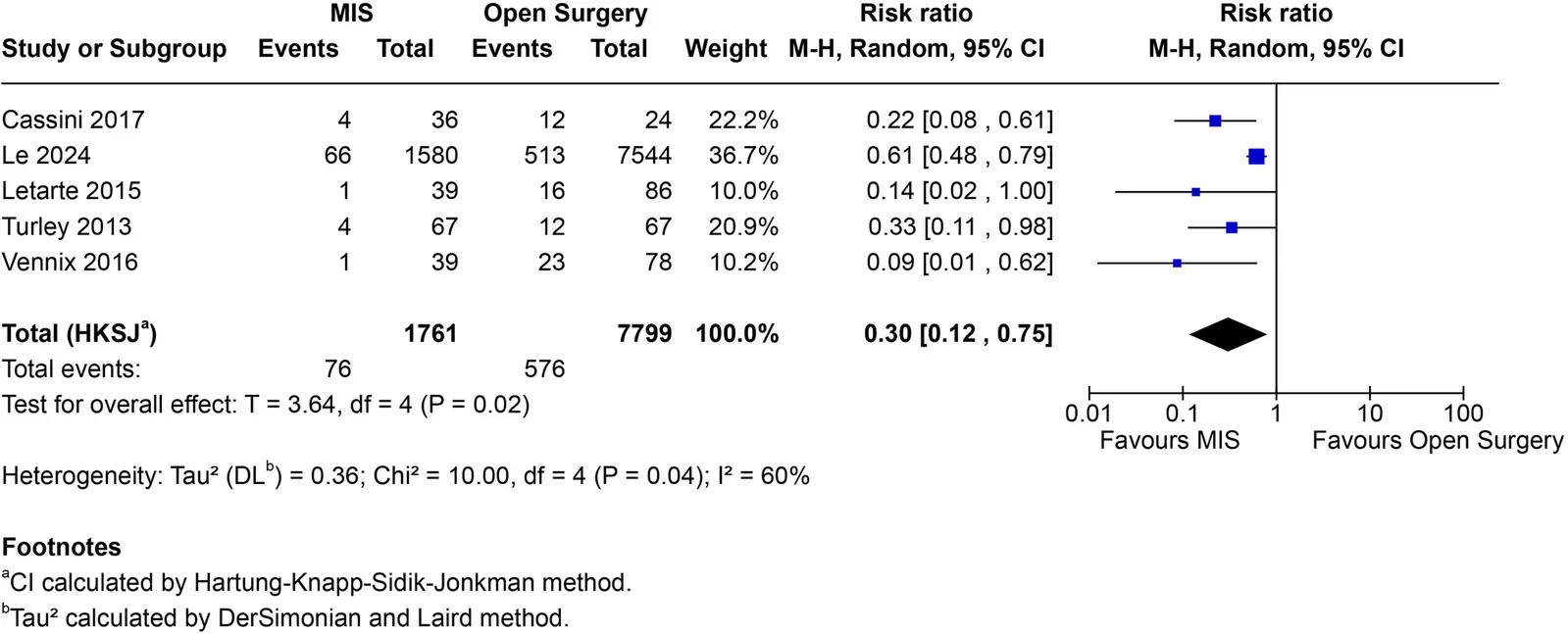
Forest plot - incisional surgical site infection. Forest plot of incisional surgical site infection (superficial and deep wound infections). Five studies (9,560 patients) demonstrated lower rates with minimally invasive surgery: RR 0.30 (95% CI 0.12–0.75; P=0.02; I²=60%). All five studies showed consistent direction favoring MIS. Le 2024 contributed 36.7% of the weight

**Fig. 9:**
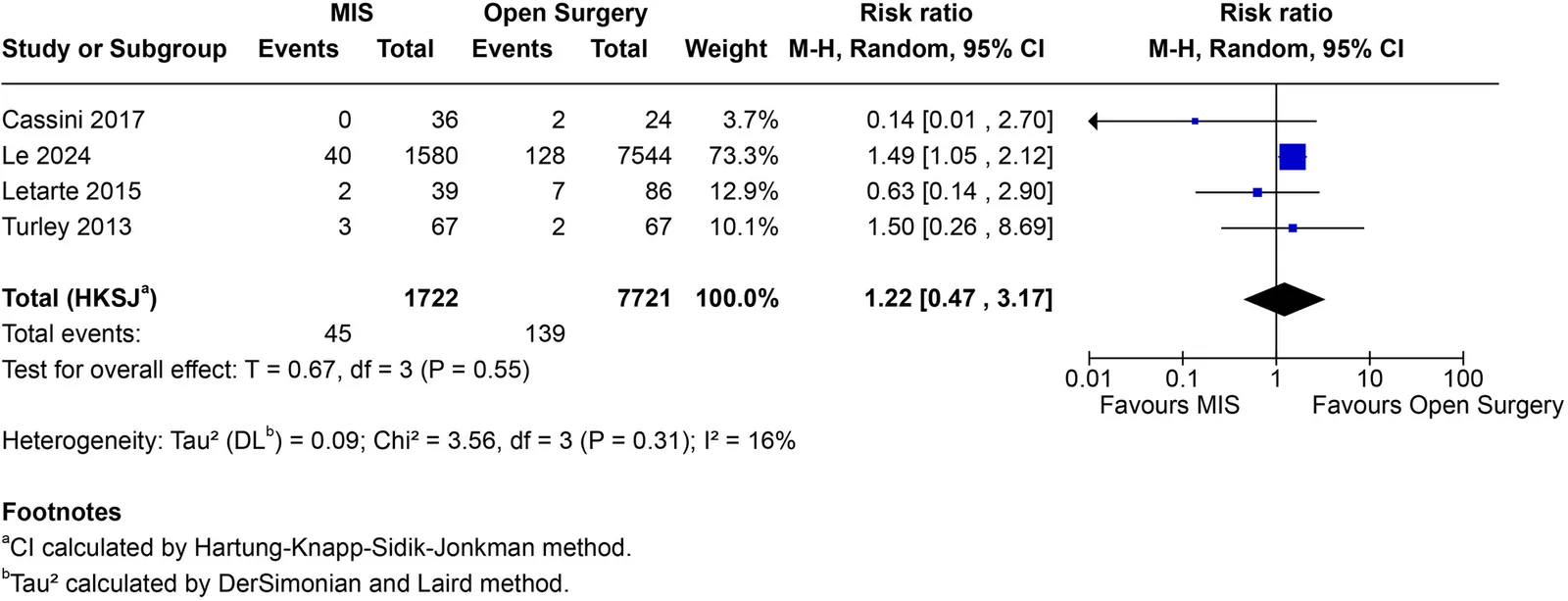
Forest plot - organ-space surgical site infection. Forest plot of organ-space surgical site infection (intra-abdominal abscess). Four studies (9,443 patients) showed no significant difference: RR 1.22 (95% CI 0.47–3.17; P=0.55; I²=16%). Low heterogeneity and wide confidence interval reflect limited event rates across studies

Surgical Site Infections. Incisional surgical site infection rates were lower with minimally invasive surgery: 76 of 1,761 (4.3%) versus 576 of 7,799 (7.4%); RR 0.30 (95% CI 0.12–0.75); *P* = 0.02; I²=60% (Fig. 8). All five studies reporting this outcome showed consistent direction favoring minimally invasive surgery, with Le et al., [20] contributing 36.7% of the analytic weight. Organ-space infections (intra-abdominal abscess) showed no significant difference: 45 of 1,722 (2.6%) versus 139 of 7,721 (1.8%); RR 1.22 (95% CI 0.47–3.17); *P* = 0.55; I²=16% (Fig. 9).

Stoma Formation. Stoma rates at index operation showed extreme heterogeneity (I²=98%) precluding meaningful pooled interpretation (Supplementary Figure S3). This variation reflected institutional philosophies regarding primary anastomosis versus Hartmann’s procedure rather than any effect of surgical approach per se. Among patients receiving primary anastomosis, protective diverting stoma rates showed no significant difference between groups: RR 0.63 (95% CI 0.07–5.46); *P* = 0.45; I²=72% (Supplementary Figure S4).

Anastomotic Leak. Only two studies (Letarte et al., [23] Vennix et al., [22]) reported anastomotic leak among patients receiving primary anastomosis, encompassing 165 patients with 5 total leak events, with differing denominator bases (Letarte whole-arm; Vennix primary-anastomosis only). The pooled estimate demonstrated extreme imprecision: RR 2.90 (95% CI 0.01–839.36); *P* = 0.25; I²=0% (Supplementary Figure S5). These sparse data preclude any meaningful conclusions regarding anastomotic safety with either surgical approach.

### Publication bias

For 30-day mortality (6 studies), visual inspection of the funnel plot showed no obvious asymmetry, though power to detect asymmetry is limited with few studies (Supplementary Figure S2). Egger’s regression test (*P* = 0.44) and Begg’s rank correlation test (*P* = 0.85) showed no statistical evidence of publication bias (Supplementary Table S8).

### Certainty of evidence

GRADE certainty assessments are presented in Table 3, with the complete evidence profile and detailed domain-by-domain explanations in Supplementary Table S9. Thirty-day mortality was rated low certainty: observational evidence began at low and was not upgraded for large effect, because the pooled estimate derived 86.9% of its weight from one database study and was no longer statistically significant when that study was excluded on leave-one-out (RR 0.61, 95% CI 0.35–1.06) [47]. Major complications was rated very low certainty due to serious risk of bias (66% of statistical weight from studies with serious confounding risk) and did not differ significantly using the conventional DerSimonian-Laird (Wald-type) random-effects interval (RR 0.63, 95% CI 0.36–1.08). Secondary outcomes including overall morbidity, length of stay, operative time, and surgical site infections were rated very low certainty due to serious risk of bias, substantial heterogeneity (I²>40%), or imprecision (confidence intervals crossing 1.0). Reoperation was rated low certainty, representing a precise null finding with no downgrades beyond the observational starting point.

**Table 3:** GRADE summary of findings

							No. of patients		Effect		Certainty	Importance
No. of studies	Study design	Risk of bias	Inconsistency	Indirectness	Imprecision	Other considerations	Minimally invasive surgery	Open surgery	Relative (95% CI)	Absolute (95% CI)		
30-day mortality												
6	non-randomised studies	not serious^a^	not serious^b^	not serious^c^	not serious^d^	none^e, f^	82/1821 (4.5%)	683/7816 (8.7%)	**RR 0.48** (0.40 to 0.59)	**45 fewer per 1**,**000** (from 52 fewer to 36 fewer)	⨁⨁◯◯ Low^a, b,c, d,e, f^	CRITICAL
Major Complications (Clavien-Dindo ≥ III)												
3	non-randomised studies	serious^g^	not serious^h^	not serious^i^	serious^q^	none^j^	21/138 (15.2%)	32/181 (17.7%)	**RR 0.63** (0.36 to 1.08)	**66 fewer per 1**,**000** (from 113 fewer to 14 more)	⨁◯◯◯ Very low^g, h,i, j,q^	CRITICAL
Overall Morbidity (Clavien-Dindo I-IV)												
5	non-randomised studies	serious^k^	serious^l^	not serious^i^	serious^m^	none^j^	98/241 (40.7%)	144/272 (52.9%)	**RR 0.70** (0.47 to 1.03)	**159 fewer per 1**,**000** (from 281 fewer to 16 more)	⨁◯◯◯ Very low^i, j,k, l,m^	CRITICAL
Unplanned Reoperation												
6	non-randomised studies	not serious^n^	not serious^h^	not serious^i^	not serious^o^	none^j^	144/1821 (7.9%)	623/7816 (8.0%)	**RR 0.98** (0.78 to 1.22)	**2 fewer per 1**,**000** (from 18 fewer to 18 more)	⨁⨁◯◯ Low^h, i,j, n,o^	CRITICAL
Length of Hospital Stay (days)												
6	non-randomised studies	not serious^n^	serious^l^	not serious^i^	not serious	none^j^	1821	7816	-	MD **2.55 days lower** (4.37 lower to 0.74 lower)	⨁◯◯◯ Very low^i, j,l, n^	IMPORTANT
Operative Time (minutes)												
6	non-randomised studies	not serious^n^	serious^l^	not serious^i^	not serious	none^d, j^	1821	7816	-	MD **18.27 min higher** (0.89 higher to 35.64 higher)	⨁◯◯◯ Very low^d, i,j, l,n^	IMPORTANT
Incisional Surgical Site Infection												
5	non-randomised studies	not serious^n^	serious^l^	not serious^i^	not serious	none^j, p^	76/1761 (4.3%)	576/7799 (7.4%)	**RR 0.30** (0.12 to 0.75)	**52 fewer per 1**,**000** (from 65 fewer to 18 fewer)	⨁◯◯◯ Very low^i, j,l, n,p^	IMPORTANT
Organ-Space Surgical Site Infection												
4	non-randomised studies	not serious^n^	not serious	not serious^i^	serious^m^	none^j^	45/1722 (2.6%)	139/7721 (1.8%)	**RR 1.22** (0.47 to 3.17)	**4 more per 1**,**000** (from 10 fewer to 39 more)	⨁◯◯◯ Very low^i, j,m, n^	IMPORTANT

CD, Clavien-Dindo; CI, confidence interval; MD, mean difference; MIS, minimally invasive surgery; RR, risk ratio; SSI, surgical site infection.

Explanations: ^a–f^30-day mortality: Not downgraded for risk of bias (87% weight from moderate-risk studies with entropy balancing/PSM), inconsistency (I²=0%), indirectness (direct PICO), imprecision (765 events, narrow CI), or publication bias (Egger *P*=0.44); not upgraded for large effect because the pooled estimate derives 86.9% of its weight from one study and is not statistically significant when that study is removed on leave-one-out (RR 0.61, 95% CI 0.35 to 1.06); certainty rated Low. ^g^Downgraded for serious risk of bias (66% weight from serious-risk studies: Paasch 46.1%, Letarte 20.0%). ^h^No downgrade; I²=0%. ^i^No downgrade; direct PICO alignment. ^j^No publication bias detected; comprehensive search. ^k^Downgraded for serious risk of bias (59% weight from serious-risk studies). ^l^Downgraded for substantial heterogeneity (I²>40%). ^m^Downgraded for imprecision; CI crosses null. ^n^Not downgraded; >85% weight from moderate-risk studies. ^o^Not downgraded; precise null finding with adequate events. ^p^No large effect upgrade despite RR<0.5 due to inconsistency (I²>50%). ^q^Major complications: the unmodified HKSJ interval (0.55 to 0.72) is anti-conservative with three studies and I²=0%; the conventional DerSimonian-Laird (Wald-type) random-effects 95% CI (0.36 to 1.08) is reported, crosses the null, and is not statistically significant

Full GRADE evidence profile with detailed domain-by-domain explanations available in Supplementary Table S9.

## Discussion

This systematic review and meta-analysis synthesizes contemporary evidence comparing emergency minimally invasive versus open left-sided colonic resection for complicated diverticulitis across 9,637 patients from six comparative studies. The findings suggest that minimally invasive surgery was associated with lower 30-day mortality, alongside shorter hospitalization despite longer operative time; the lower rate of major complications did not reach statistical significance. These associations, while clinically meaningful, must be interpreted with appropriate caution given the observational nature of all included studies and the substantial influence of one large database analysis on the pooled mortality estimate.

The observed 52% lower mortality (RR 0.48) with minimally invasive surgery was the largest effect in our analysis. The direction of effect remained favorable across the pre-specified sensitivity analyses, including restriction to propensity-score-matched studies and analysis by disease severity, although several of these subgroup estimates crossed the null. Five of the six studies showed point estimates favoring minimally invasive surgery and one showed no difference; this broad consistency, despite heterogeneous designs, is reassuring but does not exclude residual confounding. Nevertheless, the magnitude and statistical significance of this finding are substantially driven by the Le et al., [20] study, which contributed 86.9% of the weight to the mortality analysis. When this study was excluded, the point estimate remained favorable (RR 0.61) but confidence intervals widened to include the null value (*P* = 0.07). This dependency should inform clinical interpretation: while the available evidence consistently suggests benefit, definitive conclusions regarding mortality reduction await prospective confirmation.

Several mechanisms may underlie the observed associations. Minimally invasive surgery is associated with reduced surgical trauma and attenuated systemic inflammatory response, potentially relevant in patients already physiologically stressed by peritonitis or sepsis. While physiologic data were not available from the included studies, translational evidence from elective colorectal surgery meta-analyses supports this biological plausibility: Bohne et al., [48] demonstrated significantly lower postoperative interleukin-6 concentrations at 3–9 h (mean difference − 86.71 pg/ml) and reduced C-reactive protein levels at postoperative day 1 following laparoscopic compared with open colorectal resection, findings replicated across 20 randomized controlled trials with 1,131 patients. Sammour et al., [49] similarly reported significantly higher serum interleukin-6 following open versus laparoscopic colorectal surgery. Whether these inflammatory differences translate to improved clinical outcomes in emergency settings, where baseline inflammation from peritonitis is already elevated, requires prospective investigation. The lower incisional surgical site infection rate (RR 0.30) aligns with established benefits of smaller incisions and reduced tissue manipulation demonstrated in both elective and emergency colorectal surgery [50, 51]. By contrast, organ-space infection did not favor minimally invasive surgery; in the dominant study it was significantly more frequent with the minimally invasive approach (2.5% versus 1.7%) [20], a signal that warrants attention given the inflamed operative field in complicated diverticulitis. Shorter hospitalization may reflect faster recovery of gastrointestinal function and earlier mobilization, benefits enhanced by integration with enhanced recovery protocols [9, 52]. The approximately 18-minute longer operative time may be acceptable in selected patients, although this trade-off should be interpreted in light of observational confounding [20]. Technical factors such as dissection strategy and energy-device selection, which influence efficiency and safety in laparoscopic surgery generally, may also affect operative time in emergency colorectal resections [53]. 

Our findings align with previous meta-analyses examining minimally invasive versus open colorectal surgery in emergency settings more broadly [26–28]. Cirocchi et al., [26] synthesized evidence for emergency laparoscopic colectomy in complicated diverticulitis but included only 436 patients from fewer studies. This review differs from prior reviews by including a larger contemporary cohort (9,637 versus 436 patients), restricting eligibility to emergency left-sided resection for Hinchey II to IV disease, and applying an overlap algorithm for national-database studies. Warps et al., [28] examined emergency laparoscopic colorectal surgery across heterogeneous indications, limiting applicability to diverticulitis specifically. This analysis provides a focused, contemporary evidence synthesis for emergency complicated diverticulitis.

Conversion rates ranging from 4.5% to 33.7% across studies merit consideration. The lowest rate (4.5%) emerged from a Dutch multicenter study with rigorous patient selection and experienced surgeons [22], while the highest rate (33.7%) reflected contemporary real-world emergency practice in the Le et al., [20] database where surgeons attempt minimally invasive approaches broadly, converting when technical difficulties or hemodynamic instability develop. Most included studies retained converted cases within the minimally invasive arm, an approach that is conservative because, if conversion represents treatment failure, counting these patients in the minimally invasive group biases against showing benefit. Conversion handling was not uniform across studies, however, and could not be standardized, which we note as a limitation. Notably, Le et al., [20] reported that even patients requiring conversion to open surgery experienced lower odds of major adverse events compared with planned open surgery (adjusted odds ratio 0.78, *P* = 0.01), alongside reduced ostomy creation and comparable length of stay. This finding suggests that progress accomplished before conversion (including bowel mobilization, source identification, and initial dissection) may confer benefit regardless of ultimate approach, supporting the value of attempting minimally invasive surgery even when conversion becomes necessary.

Current clinical practice guidelines provide only weak recommendations regarding minimally invasive approaches for emergency complicated diverticulitis, citing limited evidence [7, 14–16]. The 2020 WSES guidelines state that laparoscopic sigmoid resection may be performed safely in selected hemodynamically stable patients at centers with established expertise, but acknowledge the evidence base is limited [7]. Our findings provide quantitative support for these recommendations while highlighting the need for careful patient selection. Recent systematic evidence on emergency minimally invasive surgery in other settings reinforces that benefit is procedure-specific and contingent on careful case selection rather than direct extrapolation from elective practice [54]. 

Heterogeneity in stoma formation and length of hospital stay merits explicit discussion. Stoma rates showed extreme heterogeneity (I²=98%), rendering pooled estimates uninterpretable. This variation reflects institutional philosophies and disease severity assessments rather than any intrinsic effect of surgical approach. Le et al., [20]. reported stoma formation in 35.8% of minimally invasive versus 84.8% of open surgery patients, reflecting aggressive primary anastomosis protocols at high-volume centers where surgeons feel comfortable with colorectal reconstruction in stable patients with limited contamination. In contrast, other studies mandated Hartmann’s procedure for all Hinchey III-IV disease regardless of approach [21, 24]. These data suggest that minimally invasive approaches may enable surgeons to consider primary anastomosis more readily due to enhanced visualization and reduced tissue trauma, but the decision to perform primary anastomosis versus Hartmann’s procedure remains driven primarily by patient stability, contamination burden, and institutional protocols rather than surgical approach per se. Importantly, the sparse data available on anastomotic leak (only 5 events across 2 studies) preclude conclusions about reconstructive safety with either approach. Length of hospital stay demonstrated substantial heterogeneity (I²=71%), reflecting diverse discharge policies, variable social support systems affecting discharge timing, and inconsistent measurement approaches (postoperative versus total hospitalization) across healthcare systems. Only one included study originated from the United States [20], and direct economic extrapolations of cost savings to other healthcare settings should be made cautiously given differing reimbursement structures, discharge criteria, and social support availability.

The interpretation of our results requires acknowledgment of several important limitations. First, all included studies were observational, and residual confounding by indication is unavoidable despite statistical adjustments employed in some studies. Patients selected for minimally invasive surgery may have been more hemodynamically stable, had less severe contamination, or been treated at centers with greater overall surgical expertise. These selection factors would bias results in favor of minimally invasive surgery independent of any true treatment effect. In the dominant study, the minimally invasive group was substantially less sick at baseline: any abnormal preoperative sepsis status (systemic inflammatory response syndrome, sepsis, or septic shock) was less frequent in the minimally invasive group (50.5% versus 67.1%), septic shock specifically was less frequent (3.9% versus 12.8%), and fewer patients were ASA class 4 or above [20]. Differences of this magnitude in baseline physiology could account for a substantial part of the observed survival association, independent of any effect of the surgical approach. Second, the substantial weight contributed by a single large database study means that our pooled estimates are sensitive to the specific population and methodology of that analysis. Third, heterogeneity in disease severity definitions, surgical techniques, and outcome measures across studies limits precision of effect estimates for some outcomes. Fourth, while translational evidence from elective surgery supports reduced inflammatory response with minimally invasive approaches, no included study directly measured perioperative inflammatory markers, limiting ability to confirm this mechanism in the emergency setting. Finally, conversion handling was not uniform across studies and could not be standardized, which may bias the attribution of outcomes between arms.

Emergency minimally invasive resection may be considered in hemodynamically stable, selected patients, by surgeons with advanced colorectal laparoscopic expertise, with a low threshold for conversion and appropriate institutional support; the data do not support a general recommendation across all patients with emergency complicated diverticulitis. Because laparoscopic colorectal outcomes depend heavily on standardized operative technique and advanced laparoscopic skill, the associations observed here are best understood as contingent on operator expertise rather than generalizable to all settings [55]. However, we caution against interpreting these observational associations as definitive evidence of causal benefit, acknowledging that residual confounding and center-level practice patterns preclude definitive causal inference. Widespread adoption as a standard of care should await confirmation from prospective registries or pragmatic randomized trials that can better control for selection bias and provide reliable estimates of treatment effect [56]. 

Future research priorities include development of prospective emergency colorectal surgery registries that systematically capture preoperative hemodynamic parameters, disease severity, surgeon experience, and granular perioperative outcomes [57]. Such registries could enable more reliable adjusted comparisons and identify patient subgroups most likely to benefit from minimally invasive approaches. Investigation of robotic-assisted surgery in emergency settings represents an emerging area warranting dedicated study as this technology becomes more widely available [16, 58]. Pragmatic randomized trials comparing operative approaches in emergency settings, while logistically challenging, would provide the strongest evidence to guide clinical practice.

## Conclusions

This systematic review and meta-analysis found that emergency minimally invasive surgery for complicated diverticulitis was associated with lower 30-day mortality (RR 0.48; low certainty evidence), an association driven by one large database study that did not remain significant when that study was excluded. A lower rate of major complications did not reach statistical significance (very low certainty). These associations may support consideration of minimally invasive resection in appropriately selected, stable patients at experienced centers. However, the substantial influence of one large database study on the mortality estimate and the inherent limitations of observational data warrant cautious interpretation. Prospective validation through registries or pragmatic trials is needed before recommending widespread adoption.

## Supplementary Information

Supplementary Material 1. Supplementary materials, comprising Supplementary Text S1 to S3, Supplementary Tables S1 to S9 (complete search strategy, excluded studies with reasons, ROBINS-I risk-of-bias assessment at the outcome level, sensitivity and subgroup analyses, outcome-definition harmonization, median-to-mean conversion details, inter-rater reliability for study selection, publication-bias assessment, and the GRADE evidence profile), and Supplementary Figures S1 to S5 with legends. Supplementary Figure S1: Forest plot of 30-day mortality sensitivity analysis excluding Le 2024. Five studies (513 patients) yielded RR 0.61 (95% CI 0.35–1.06; *P*=0.07; I²=0%). Point estimate maintains directional consistency without achieving statistical significance, demonstrating the influence of Le 2024 on pooled conclusions. Supplementary Figure S2: Funnel plot for publication bias assessment of 30-day mortality. Standard error plotted against log risk ratio for all six studies. No obvious asymmetry observed, though power to detect asymmetry is limited with only six studies. Le 2024 positioned at apex (largest, most precise study). Supplementary Figure S3: Forest plot of any stoma at index operation. Four studies showed extreme heterogeneity (I²=98%) precluding meaningful interpretation: RR 0.61 (95% CI 0.27–1.39; *P*=0.15). Stoma formation reflects institutional protocols and disease severity rather than surgical approach. Supplementary Figure S4: Forest plot of protective diverting stoma among primary anastomosis patients. Three studies (168 patients) showed no significant difference: RR 0.63 (95% CI 0.07–5.46; *P*=0.45; I²=72%). Substantial heterogeneity reflects variable surgeon thresholds for diversion. Supplementary Figure S5: Forest plot of anastomotic leak. Two studies (165 patients) with 5 total leaks demonstrated extreme imprecision: RR 2.90 (95% CI 0.01–839.36; *P*=0.25). Denominator bases differed (Letarte whole-arm; Vennix primary-anastomosis only). Insufficient data preclude meaningful conclusions about anastomotic safety.

Supplementary Material 2.

## Data Availability

The systematic review protocol was prospectively registered with PROSPERO (CRD420251142546) and is available at https://www.crd.york.ac.uk/prospero/. Extracted datasets generated during this review, including study-level data and meta-analysis outputs, are available from the corresponding author upon reasonable request. Complete search strategies, risk of bias assessments, detailed statistical analyses, and additional results are provided in theSupplementary Materials accompanying this manuscript. The statistical code used for meta-analyses is available in the study protocol.

## Abbreviations

ASA: American Society of Anesthesiologists
BMI: Body mass index
CD: Clavien-Dindo
CI: Confidence interval
EAES: European Association for Endoscopic Surgery
ESCP: European Society of Coloproctology
GRADE: Grading of Recommendations Assessment, Development and Evaluation
HKSJ: Hartung-Knapp-Sidik-Jonkman
MD: Mean difference
MIS: Minimally invasive surgery
NNT: Number needed to treat
NR: Not reported
NSQIP: National Surgical Quality Improvement Program
PICO: Population, Intervention, Comparator, Outcome
PRISMA: Preferred Reporting Items for Systematic Reviews and Meta-Analyses
PROSPERO: International Prospective Register of Systematic Reviews
PSM: Propensity score matching
ROBINS-I: Risk of Bias in Non-randomized Studies of Interventions
RR: Risk ratio
SSI: Surgical site infection
WSES: World Society of Emergency Surgery
