# Supplementary material for: Emergency minimally invasive versus open left-sided colonic resection for complicated diverticulitis: a systematic review and meta-analysis
Source: BMC Surg. 2026 Jul 21;26:695. doi: 10.1186/s12893-026-04043-3 (PMC13673721; doi:10.1186/s12893-026-04043-3)
Supplement: Supplementary file 1 [file 12893_2026_4043_MOESM1_ESM.docx]

**SUPPLEMENTARY MATERIALS**

Emergency Minimally Invasive versus Open Left-Sided Colonic Resection for Complicated Diverticulitis: A Systematic Review and Meta-Analysis

**SUPPLEMENTARY TABLES**

**Supplementary Table S1. Complete Search Strategy**

**Table S1a. Search Strategy Summary**

| **Database** | **Search Date** | **Filters** | **Results** |
| --- | --- | --- | --- |
| PubMed/MEDLINE | July 2025 | None | 293 |
| Scopus | July 2025 | None | 576 |
| Cochrane CENTRAL | July 2025 | Trials | 38 |
| ClinicalTrials.gov | July 2025 | All studies | 35 |
| Total identified |  |  | 942 |
| Duplicates removed |  |  | 258 |
| Unique records screened |  |  | 684 |

**Table S1b. PubMed/MEDLINE Search Strategy**

| **Line** | **Search Terms** |
| --- | --- |
| #1 | "diverticulitis"[MeSH Terms] OR "diverticulitis, colonic"[MeSH Terms] OR diverticulitis[Title/Abstract] OR "diverticular disease"[Title/Abstract] |
| #2 | "laparoscopy"[MeSH Terms] OR "minimally invasive surgical procedures"[MeSH Terms] OR laparoscop*[Title/Abstract] OR "minimally invasive"[Title/Abstract] OR MIS[Title/Abstract] OR robotic[Title/Abstract] |
| #3 | "emergencies"[MeSH Terms] OR "emergency medical services"[MeSH Terms] OR emergency[Title/Abstract] OR urgent[Title/Abstract] OR acute[Title/Abstract] |
| #4 | "colectomy"[MeSH Terms] OR "digestive system surgical procedures"[MeSH Terms] OR colectomy[Title/Abstract] OR resection[Title/Abstract] OR "surgical procedures, operative"[MeSH Terms] |
| #5 | #1 AND #2 AND #3 AND #4 |

Search date: July 2025 | Filters: None (no language restrictions) | Results: 293 citations

**Table S1c. Scopus Search Strategy**

| **Line** | **Search Terms** |
| --- | --- |
| #1 | TITLE-ABS-KEY (diverticulitis OR "diverticular disease") |
| #2 | TITLE-ABS-KEY (laparoscop* OR "minimally invasive" OR MIS OR robotic) |
| #3 | TITLE-ABS-KEY (emergency OR urgent OR acute OR complicated) |
| #4 | TITLE-ABS-KEY (colectomy OR resection OR surgery OR operative) |
| #5 | #1 AND #2 AND #3 AND #4 |
| #6 | TITLE-ABS-KEY ("complicated diverticulitis" AND (laparoscop* OR "minimally invasive") AND (emergency OR urgent) AND (colectomy OR resection)) |

Search date: July 2025 | Filters: None | Results: 576 citations (555 primary + 21 refined)

**Table S1d. Cochrane CENTRAL Search Strategy**

| **Line** | **Search Terms** |
| --- | --- |
| #1 | MeSH descriptor: [Diverticulitis] explode all trees |
| #2 | MeSH descriptor: [Laparoscopy] explode all trees |
| #3 | MeSH descriptor: [Emergencies] explode all trees |
| #4 | #1 AND #2 AND #3 |

Search date: July 2025 | Filters: Trials | Results: 38 citations

**Table S1e. ClinicalTrials.gov Search Strategy**

| **Field** | **Search Terms** |
| --- | --- |
| Condition | diverticulitis |
| Intervention | laparoscopic OR minimally invasive |
| Other terms | emergency OR urgent OR acute |
| Status | All studies |

Search date: July 2025 | Results: 35 trial registrations

**Supplementary Table S2. Excluded Studies with Reasons**

| **Study** | **Year** | **Reason for Exclusion** |
| --- | --- | --- |
| NSQIP-based studies with temporal overlap with Le 2024 |  |  |
| Amodu et al.[1] | 2024 | NSQIP 2015–2019; 100% contained within Le 2024 (2015–2020) |
| Moghadamyeghaneh et al.[2] | 2021 | NSQIP 2012–2017; partial overlap with Le 2024 (2015–2017) |
| Lee et al.[3] | 2020 | NSQIP 2012–2017; partial overlap with Le 2024 (2015–2017) |
| Esparza Monzavi et al.[4] | 2021 | NSQIP 2012–2017; partial overlap with Le 2024 (2015–2017) |
| Hajirawala et al.[5] | 2022 | NSQIP 2013–2018; partial overlap with Le 2024 (2015–2018) |
| Early surgical era |  |  |
| Rea et al.[6] | 2012 | Study period 2003–2007; administrative database (Nationwide Inpatient Sample) without per-patient outcome data compatible with 2×2 extraction, and a 55% conversion rate reflecting an earlier surgical era |
| Other exclusions |  |  |
| Vennix et al.[7] (LADIES trial) | 2015 | RCT of lavage vs resection; no MIS vs open resection comparison |
| Schultz et al.[8] (SCANDIV) | 2015 | RCT of lavage vs resection; no MIS vs open resection comparison |
| Thornell et al.[9] (DILALA) | 2016 | RCT of lavage vs Hartmann's; MIS in both arms |
| Multiple case series | Various | No comparator group |
| Mixed elective/emergency | Various | No separate emergency outcome reporting |

Selection algorithm for overlapping database studies: When multiple studies used overlapping populations from the same database (e.g., NSQIP), we retained only the largest, most recent study with the most rigorous statistical adjustment. Le et al. (2024) was selected because it: (1) had the largest sample size (Le 2024 reports 9,194 in its flowchart and text, while the analytic denominators used for our pooled outcomes are 9,124, comprising 7,544 open and 1,580 minimally invasive cases), (2) was the most recent analysis (published 2024), and (3) employed entropy balancing, which provides more robust confounding adjustment than standard propensity score matching.

**References for excluded studies:**

Amodu LI, Hakmi H, Sohail AH, Akerman M, Petrone P, Halpern DK, Sonoda T (2024) Laparoscopic Hartmann's procedure for complicated diverticulitis is associated with lower superficial surgical site infections compared to open surgery with similar other outcomes: a NSQIP-based, propensity score matched analysis. Eur J Trauma Emerg Surg 50(5):2097-2103. https://doi.org/10.1007/s00068-024-02661-1

Moghadamyeghaneh Z, Talus H, Fitzgerald S, Muthusamy M, Stamos MJ, Roudnitsky V (2021) Outcomes of minimally invasive colectomy for perforated diverticulitis. Am Surg 87(4):561-567. https://doi.org/10.1177/0003134820950295

Lee YF, Brown RF, Battaglia M, Cleary RK (2020) Laparoscopic versus open emergent sigmoid resection for perforated diverticulitis. J Gastrointest Surg 24(5):1173-1182. https://doi.org/10.1007/s11605-019-04490-9

Esparza Monzavi CA, Naffouje SA, Chaudhry V, Nordenstam J, Mellgren A, Gantt G (2021) Open vs minimally invasive approach for emergent colectomy in perforated diverticulitis. Dis Colon Rectum 64(3):319-327. https://doi.org/10.1097/DCR.0000000000001805

Hajirawala LN, Moreci R, Leonardi C, Bevier-Rawls ER, Orangio GR, Davis KG, Barton JS, Klinger AL (2022) Laparoscopic colectomy for acute diverticulitis in the urgent setting is associated with similar outcomes to open. Am Surg 88(5):901-907. https://doi.org/10.1177/00031348211054553

Rea JD, Herzig DO, Diggs BS, Cone MM, Lu KC (2012) Use and outcomes of emergent laparoscopic resection for acute diverticulitis. Am J Surg 203(5):639-643. https://doi.org/10.1016/j.amjsurg.2012.01.004

Vennix S, Musters GD, Mulder IM, Swank HA, Consten EC, Belgers EH, van Geloven AA, Gerhards MF, Govaert MJ, van Grevenstein WM, Hoofwijk AG, Kruyt PM, Nienhuijs SW, Boermeester MA, Vermeulen J, van Dieren S, Lange JF, Bemelman WA; Ladies trial colloborators (2015) Laparoscopic peritoneal lavage or sigmoidectomy for perforated diverticulitis with purulent peritonitis: a multicentre, parallel-group, randomised, open-label trial. Lancet 386(10000):1269-1277. https://doi.org/10.1016/S0140-6736(15)61168-0

Schultz JK, Yaqub S, Wallon C, Blecic L, Forsmo HM, Folkesson J, Buchwald P, Körner H, Dahl FA, Øresland T; SCANDIV Study Group (2015) Laparoscopic lavage vs primary resection for acute perforated diverticulitis: the SCANDIV randomized clinical trial. JAMA 314(13):1364-1375. https://doi.org/10.1001/jama.2015.12076

Thornell A, Angenete E, Bisgaard T, Bock D, Burcharth J, Heath J, Pommergaard HC, Rosenberg J, Stilling N, Skullman S, Haglind E (2016) Laparoscopic lavage for perforated diverticulitis with purulent peritonitis: a randomized trial. Ann Intern Med 164(3):137-145. https://doi.org/10.7326/M15-1210

**Supplementary Table S3. ROBINS-I Risk of Bias Assessment at Outcome Level**

**30-Day Mortality**

| **Study** | **Confounding** | **Selection** | **Classification** | **Deviations** | **Missing Data** | **Measurement** | **Reporting** | **Overall** |
| --- | --- | --- | --- | --- | --- | --- | --- | --- |
| Le 2024 | Moderate | Low | Low | Low | Low | Low | Low | Moderate |
| Turley 2013 | Moderate | Low | Low | Low | Low | Low | Low | Moderate |
| Vennix 2016 | Moderate | Low | Low | Low | Low | Low | Low | Moderate |
| Letarte 2015 | Serious | Low | Low | Low | Low | Low | Low | Serious |
| Cassini 2017 | Serious | Low | Low | Low | Moderate | Low | Low | Serious |
| Paasch 2022 | Serious | Low | Low | Low | Low | Low | Low | Serious |

**Major Complications (Clavien-Dindo ≥III)**

| **Study** | **Confounding** | **Selection** | **Classification** | **Deviations** | **Missing Data** | **Measurement** | **Reporting** | **Overall** |
| --- | --- | --- | --- | --- | --- | --- | --- | --- |
| Vennix 2016 | Moderate | Low | Low | Low | Low | Moderate | Low | Moderate |
| Letarte 2015 | Serious | Low | Low | Low | Low | Moderate | Low | Serious |
| Paasch 2022 | Serious | Low | Low | Low | Low | Moderate | Low | Serious |

Note: Le 2024 excluded (composite "major adverse events" not equivalent to Clavien-Dindo grading). Turley 2013 excluded (no Clavien-Dindo classification). Cassini 2017 excluded (event-level complications, not per-patient; 16/60 patients had multiple complications).

**Overall Morbidity**

| **Study** | **Confounding** | **Selection** | **Classification** | **Deviations** | **Missing Data** | **Measurement** | **Reporting** | **Overall** |
| --- | --- | --- | --- | --- | --- | --- | --- | --- |
| Turley 2013 | Moderate | Low | Low | Low | Low | Moderate | Low | Moderate |
| Vennix 2016 | Moderate | Low | Low | Low | Low | Moderate | Low | Moderate |
| Letarte 2015 | Serious | Low | Low | Low | Low | Moderate | Low | Serious |
| Cassini 2017 | Serious | Low | Low | Low | Moderate | Moderate | Low | Serious |
| Paasch 2022 | Serious | Low | Low | Low | Low | Moderate | Low | Serious |

**Reoperation**

| **Study** | **Confounding** | **Selection** | **Classification** | **Deviations** | **Missing Data** | **Measurement** | **Reporting** | **Overall** |
| --- | --- | --- | --- | --- | --- | --- | --- | --- |
| Le 2024 | Moderate | Low | Low | Low | Low | Low | Low | Moderate |
| Turley 2013 | Moderate | Low | Low | Low | Low | Low | Low | Moderate |
| Vennix 2016 | Moderate | Low | Low | Low | Low | Low | Low | Moderate |
| Letarte 2015 | Serious | Low | Low | Low | Low | Low | Low | Serious |
| Cassini 2017 | Serious | Low | Low | Low | Moderate | Low | Low | Serious |
| Paasch 2022 | Serious | Low | Low | Low | Low | Low | Low | Serious |

**Length of Hospital Stay**

| **Study** | **Confounding** | **Selection** | **Classification** | **Deviations** | **Missing Data** | **Measurement** | **Reporting** | **Overall** |
| --- | --- | --- | --- | --- | --- | --- | --- | --- |
| Le 2024 | Moderate | Low | Low | Low | Low | Low | Low | Moderate |
| Turley 2013 | Moderate | Low | Low | Low | Low | Low | Low | Moderate |
| Vennix 2016 | Moderate | Low | Low | Low | Low | Low | Low | Moderate |
| Letarte 2015 | Serious | Low | Low | Low | Low | Low | Low | Serious |
| Cassini 2017 | Serious | Low | Low | Low | Moderate | Low | Low | Serious |
| Paasch 2022 | Serious | Low | Low | Low | Low | Low | Low | Serious |

Note: Paasch 2022 reported LOS for survivors only (MIS: n=52; Open: n=12).

**Operative Time**

| **Study** | **Confounding** | **Selection** | **Classification** | **Deviations** | **Missing Data** | **Measurement** | **Reporting** | **Overall** |
| --- | --- | --- | --- | --- | --- | --- | --- | --- |
| Le 2024 | Moderate | Low | Low | Low | Low | Low | Low | Moderate |
| Turley 2013 | Moderate | Low | Low | Low | Low | Low | Low | Moderate |
| Vennix 2016 | Moderate | Low | Low | Low | Low | Low | Low | Moderate |
| Letarte 2015 | Serious | Low | Low | Low | Low | Low | Low | Serious |
| Cassini 2017 | Serious | Low | Low | Low | Moderate | Low | Low | Serious |
| Paasch 2022 | Serious | Low | Low | Low | Low | Low | Low | Serious |

Note: All studies used skin incision to closure definition. Turley 2013 reported unmatched cohort operative times.

**Incisional Surgical Site Infection**

| **Study** | **Confounding** | **Selection** | **Classification** | **Deviations** | **Missing Data** | **Measurement** | **Reporting** | **Overall** |
| --- | --- | --- | --- | --- | --- | --- | --- | --- |
| Le 2024 | Moderate | Low | Low | Low | Low | Low | Low | Moderate |
| Turley 2013 | Moderate | Low | Low | Low | Low | Low | Low | Moderate |
| Vennix 2016 | Moderate | Low | Low | Low | Low | Low | Low | Moderate |
| Letarte 2015 | Serious | Low | Low | Low | Low | Moderate | Low | Serious |
| Cassini 2017 | Serious | Low | Low | Low | Moderate | Moderate | Low | Serious |

Note: Paasch 2022 excluded (incisional SSI not separately reported). CDC definitions used: Le 2024 and Turley 2013 (NSQIP standardized); Vennix 2016 (prospective); Letarte 2015 and Cassini 2017 (retrospective ascertainment).

**Organ-Space Surgical Site Infection**

| **Study** | **Confounding** | **Selection** | **Classification** | **Deviations** | **Missing Data** | **Measurement** | **Reporting** | **Overall** |
| --- | --- | --- | --- | --- | --- | --- | --- | --- |
| Le 2024 | Moderate | Low | Low | Low | Low | Low | Low | Moderate |
| Turley 2013 | Moderate | Low | Low | Low | Low | Low | Low | Moderate |
| Letarte 2015 | Serious | Low | Low | Low | Low | Moderate | Low | Serious |
| Cassini 2017 | Serious | Low | Low | Low | Moderate | Moderate | Low | Serious |

Note: Vennix 2016 and Paasch 2022 excluded (organ-space SSI not separately reported). Cassini 2017 had zero events in MIS arm (0/36 vs 2/24).

**Domain-Level Rationale:**

Confounding: Studies with propensity score matching or entropy balancing (Le, Turley, Vennix) were rated "Moderate" acknowledging residual confounding is inevitable. Studies with limited adjustment (Paasch: 3 covariates via MVA) or no adjustment (Letarte, Cassini) were rated "Serious."

Selection of Participants: All studies clearly defined inclusion criteria for emergency complicated diverticulitis; rated "Low" across all studies.

Classification of Interventions: Surgical approach (MIS vs open) was clearly defined and recorded prospectively; rated "Low" across all studies.

Deviations from Intended Interventions: Analyses followed each source study's reported arm definitions; most studies retained converted cases within the MIS group, although conversion handling was not uniform across studies; rated "Low."

Missing Data: Most studies had complete outcome data; Cassini 2017 had some missing follow-up data, rated "Moderate."

**Measurement of Outcomes:**

Mortality and reoperation: Objectively measured ("Low" for all studies).

Major complications and overall morbidity: Clavien-Dindo grading requires clinical judgment; rated "Moderate" for institutional cohorts due to potential inter-observer variability.

Length of stay and operative time: Objectively measured from medical records ("Low" for all studies).

Surgical site infections: NSQIP studies (Le 2024, Turley 2013) and prospective studies (Vennix 2016) used standardized CDC definitions ("Low"). Retrospective institutional cohorts (Letarte 2015, Cassini 2017) relied on chart review without standardized surveillance ("Moderate").

Selection of Reported Results: All studies reported pre-specified outcomes; no evidence of selective reporting; rated "Low."

**Supplementary Table S4. Sensitivity and Subgroup Analyses**

**Mortality Sensitivity Analyses**

| **Analysis** | **Studies** | **n** | **RR (95% CI)** | **P-value** | **I²** |
| --- | --- | --- | --- | --- | --- |
| Primary analysis (all studies) | 6 | 9,637 | 0.48 (0.40–0.59) | 0.0002 | 0% |
| Excluding Le 2024 | 5 | 513 | 0.61 (0.35–1.06) | 0.07 | 0% |
| Advanced adjustment only (Le, Vennix, Turley) | 3 | 9,375 | 0.49 (0.40–0.60) | <0.001 | 0% |
| Unadjusted studies only | 3 | 262 | 0.58 (0.28–1.20) | 0.11 | 0% |
| Studies ≥2015 only | 4 | 9,378 | 0.48 (0.39–0.59) | <0.001 | 0% |
| Hinchey III–IV only | 3 | 254 | 0.60 (0.33–1.10) | 0.09 | 0% |

**Major Complications Method Note**

Major-complications sensitivity analyses based on the earlier unmodified HKSJ model were removed after the final method decision, because the unmodified interval was anti-conservative with three studies and I²=0%. The final Wald-type estimate (RR 0.63, 95% CI 0.36–1.08; P=0.10) is reported in Figure 3, main Table 3, and Supplementary Table S9.

**Leave-One-Out Analysis for Mortality**

| **Study Excluded** | **Remaining RR (95% CI)** | **P-value** | **I²** | **Change from Primary** |
| --- | --- | --- | --- | --- |
| Cassini 2017 | 0.47 (0.42–0.53) | <0.0001 | 0% | −2% |
| Le 2024 | 0.61 (0.35–1.06) | 0.07 | 0% | +27%, CI crosses 1.0 |
| Letarte 2015 | 0.49 (0.39–0.61) | 0.0008 | 0% | +2% |
| Paasch 2022 | 0.49 (0.38–0.62) | 0.001 | 0% | +2% |
| Turley 2013 | 0.48 (0.38–0.60) | 0.0009 | 0% | 0% |
| Vennix 2016 | 0.48 (0.38–0.61) | 0.0009 | 0% | 0% |

Note: Exclusion of Le 2024 is the only analysis that substantially alters conclusions, with the point estimate changing from RR 0.48 to RR 0.61 (+27%) and 95% CI widening to cross 1.0 (P changes from 0.0002 to 0.07). All other studies have minimal influence (≤2% change). This pattern reflects Le 2024's dominant weight (86.9%) due to its large sample size. The consistent direction of effect across all leave-one-out analyses supports the robustness of the observed association, while the dependency on Le 2024 for statistical significance should inform clinical interpretation.

**Supplementary Table S5. Outcome Definition Harmonization**

| **Outcome** | **Definition Used** | **Studies Using This Definition** | **Notes** |
| --- | --- | --- | --- |
| 30-day mortality | Death within 30 days of surgery | Le, Turley, Vennix, Letarte | Primary definition |
| In-hospital mortality | Death during index hospitalization | Cassini, Paasch | Used when 30-day not reported |
| Major complications | Clavien-Dindo grade III–IV (excluding death) | Vennix, Letarte, Paasch | Standardized grading system |
| Overall morbidity | Clavien-Dindo grade I–IV (excluding death) | Turley, Vennix, Letarte, Cassini, Paasch | Any postoperative complication |
| Reoperation | Unplanned return to OR during index hospitalization | All 6 studies | Consistent definition |
| Length of stay | Days from surgery to discharge | All 6 studies | Median converted to mean where needed |
| Operative time | Minutes from incision to closure | All 6 studies | Median converted to mean where needed |
| Incisional SSI | Superficial + deep wound infection | Cassini, Le, Letarte, Turley, Vennix | CDC definition where specified |
| Organ-space SSI | Intra-abdominal abscess | Cassini, Le, Letarte, Turley | Post-operative abscess requiring drainage |
| Anastomotic leak | Clinical or radiological leak | Letarte, Vennix | Letarte whole-arm; Vennix primary-anastomosis only; sparse events, interpret cautiously |

**Exclusions from specific outcomes:**

Le 2024: Excluded from major complications and overall morbidity due to non-comparable composite "major adverse events" definition

Turley 2013: Excluded from major complications (did not use Clavien-Dindo grading)

Cassini 2017: Excluded from pooled major complications and anastomotic leak because complications were reported at the event level rather than as clean per-patient Clavien-Dindo ≥III (excluding death)

**Supplementary Table S6. Median-to-Mean Conversion Details**

For studies reporting medians with interquartile ranges (IQR) or ranges, we estimated means and standard deviations using the methods of Wan et al. (BMC Med Res Methodol. 2014;14:135) and Hozo et al. (BMC Med Res Methodol. 2005;5:13).

**Length of Stay (days)**

| **Study** | **Reported Values** | **Converted Mean (SD)** | **Method** |
| --- | --- | --- | --- |
| Le 2024 | MIS: 7 [5, 11]; Open: 9 [6, 13] | MIS: 7.67 (4.44); Open: 9.33 (5.19) | Wan (IQR) |
| Turley 2013 | MIS: 6 [5–11]; Open: 8 [6–11] (matched cohort) | MIS: 7.33 (4.44); Open: 8.33 (3.70) | Wan (IQR) |
| Vennix 2016 | MIS: 7 (5–13); Open: 9 (7–14) | MIS: 8.33 (5.93); Open: 10.0 (5.19) | Wan (IQR) |
| Letarte 2015 | MIS: 9.7 ± 6.5; Open: 13.3 ± 8.3 | MIS: 9.7 (6.5); Open: 13.3 (8.3) | As reported |
| Cassini 2017 | MIS: 8.1 (4–30); Open: 12.8 (5–23)ᵃ | MIS: 8.1 (6.5); Open: 12.8 (4.5) | Mean (range); SD estimated |
| Paasch 2022 | MIS: 9 [7–14]; Open: 17 [11–21]ᵇ | MIS: 10.0 (5.19); Open: 16.33 (7.41) | Wan (IQR); survivors only |

ᵃCassini 2017 reported mean (range), not median (range). ᵇPaasch 2022 LOS reported for survivors only (MIS: n=52; Open: n=12).

**Operative Time (minutes)**

| **Study** | **Reported Values** | **Converted Mean (SD)** | **Method** |
| --- | --- | --- | --- |
| Le 2024 | MIS: 158 [119, 210]; Open: 128 [95, 172] | MIS: 162.3 (67.4); Open: 131.7 (57.0) | Wan (IQR) |
| Turley 2013 | MIS: 132 ± 64; Open: 138 ± 62 (matched cohort, n=67 per arm) | MIS: 132 (64); Open: 138 (62) | As reported |
| Vennix 2016 | MIS: 127 (105–159); Open: 96.5 (87–120) | MIS: 130.3 (40.0); Open: 101.2 (24.4) | Wan (IQR) |
| Letarte 2015 | MIS: 273.6 ± 60.3; Open: 241.8 ± 65.5 | MIS: 273.6 (60.3); Open: 241.8 (65.5) | As reported |
| Cassini 2017 | MIS: 170.4 (120–240); Open: 169.6 (120–255)ᵃ | MIS: 170.4 (30); Open: 169.6 (34) | Mean (range); SD estimated |
| Paasch 2022 | MIS: 120 [90–162]; Open: 100 [69–156] | MIS: 124.0 (53.3); Open: 108.3 (64.4) | Wan (IQR) |

ᵃCassini 2017 reported mean (range), not median (range). SD estimated from range using Range/4 approximation.

**Supplementary Table S7. Inter-Rater Reliability for Study Selection**

| **Stage** | **Metric** | **Value** | **Interpretation** |
| --- | --- | --- | --- |
| Title/Abstract Screening | Cohen's kappa (κ) | 0.92 | Almost perfect agreement |
|  | Percent agreement | 96.8% |  |
|  | Discordant records | 30/942 | Resolved by discussion |
| Full-Text Review | Cohen's kappa (κ) | 0.88 | Almost perfect agreement |
|  | Percent agreement | 94.6% |  |
|  | Discordant articles | 6/112 | Resolved by discussion |
| Data Extraction | Intraclass correlation (ICC) | 0.97 | Excellent reliability |
|  | Discrepancies | 12 data points | Resolved by source verification |

**Interpretation of kappa values:**

0.81–1.00: Almost perfect agreement

0.61–0.80: Substantial agreement

0.41–0.60: Moderate agreement

0.21–0.40: Fair agreement

0.00–0.20: Slight agreement

**Supplementary Table S8. Publication Bias Assessment**

**Egger's Regression Test**

| **Outcome** | **Studies** | **Intercept** | **95% CI** | **P-value** |
| --- | --- | --- | --- | --- |
| 30-day mortality | 6 | 0.32 | −1.08 to 1.72 | 0.44 |
| Major complications | 3 | — | — | Not calculable (n<6) |
| Overall morbidity | 5 | −1.23 | −4.56 to 2.10 | 0.38 |
| Reoperation | 6 | 0.18 | −1.89 to 2.25 | 0.82 |

**Begg's Rank Correlation Test**

| **Outcome** | **Studies** | **Kendall's tau** | **P-value** |
| --- | --- | --- | --- |
| 30-day mortality | 6 | 1.00 | 0.85 |
| Major complications | 3 | — | Not calculable (n<6) |
| Overall morbidity | 5 | −0.20 | 0.46 |
| Reoperation | 6 | 0.07 | 0.85 |

Interpretation: No statistical evidence of publication bias was detected for any outcome with sufficient studies for analysis. However, power to detect asymmetry is limited with only 6 studies, and these tests should be interpreted with caution. Visual inspection of funnel plots (Supplementary Figure S2) showed no obvious asymmetry.

**SUPPLEMENTARY TEXT**

**Supplementary Text S1. Definition of "Emergency Surgery"**

For this systematic review, we defined “emergency surgery” as an unplanned surgical procedure performed during the index hospitalization for acute complicated diverticulitis. This definition encompasses two scenarios:

Immediate surgery (within 24 hours): Patients requiring urgent operative intervention due to hemodynamic instability, diffuse peritonitis, or failure to respond to initial resuscitation.

Urgent surgery after initial management: Patients who initially undergo non-operative management (antibiotics, percutaneous drainage) but subsequently require surgery during the same hospitalization due to treatment failure, clinical deterioration, or persistent/recurrent symptoms.

This pragmatic definition reflects real-world clinical practice and was used by most included studies. We excluded purely elective operations (scheduled surgery after recovery from acute episode) and semi-elective procedures (planned early surgery during controlled conditions).

The rationale for this inclusive definition is that both immediate and urgent surgery share the key features that distinguish emergency from elective surgery: acute inflammatory changes, potential for contamination, and limited time for optimization. This approach maximizes the generalizability of our findings to clinical practice.

Studies varied in their specific terminology:

Le 2024: “emergent colectomy” (ICD-10 procedure codes with emergency admission)

Turley 2013: “emergency treatment” (NSQIP emergency case flag)

Vennix 2016: “acute sigmoidectomy” (within 7 days of admission)

Letarte 2015: “emergent surgical treatment” (during index admission)

Cassini 2017: “emergency surgery” (Hinchey III–IV requiring immediate intervention)

Paasch 2022: “emergency resection” (urgent indication, not elective)

**Supplementary Text S2. Statistical Methods Detail**

**Random-Effects Model with HKSJ Adjustment**

We employed the Hartung-Knapp-Sidik-Jonkman (HKSJ) adjustment for the confidence intervals of our random-effects meta-analyses, with a single exception. For one outcome (major complications; three studies with I²=0%), the unmodified HKSJ interval became narrower than the conventional interval, the recognised anti-conservative behaviour of HKSJ when the number of studies is small and the between-study heterogeneity estimate is zero; for that outcome we instead reported the conventional DerSimonian-Laird (Wald-type) random-effects confidence interval (RR 0.63, 95% CI 0.36 to 1.08; P=0.10). The HKSJ method modifies the standard DerSimonian-Laird approach by using a t-distribution rather than a normal distribution for the confidence interval calculation and by incorporating an adjusted variance estimator.

The HKSJ method provides several advantages in meta-analyses with few studies:

More appropriate confidence interval coverage (closer to nominal 95%)

Reduced false-positive rates compared to DerSimonian-Laird

More conservative estimates when heterogeneity is uncertain

For dichotomous outcomes, we calculated risk ratios using the Mantel-Haenszel method for pooling with the DerSimonian-Laird estimator for between-study variance (τ²), followed by HKSJ adjustment for the confidence interval.

**Handling Zero Events**

For studies with zero events in one arm (e.g., zero mortality in the MIS group), we applied a 0.5 continuity correction to all cells of the 2×2 table. This approach:

Allows inclusion of studies with zero events in one arm

Provides slightly conservative effect estimates

Is the default approach in RevMan and most meta-analysis software

Studies with zero events in both arms were excluded from that specific outcome analysis, as they provide no information about the relative effect.

**Heterogeneity Assessment**

We assessed statistical heterogeneity using:

Cochran's Q test: Tests whether observed differences between studies exceed sampling error. P<0.10 considered significant given low power.

I² statistic: Proportion of variability due to heterogeneity rather than chance.

0–40%: Might not be important

30–60%: Moderate heterogeneity

50–90%: Substantial heterogeneity

75–100%: Considerable heterogeneity

We did not use prediction intervals due to the small number of studies (prediction intervals require ≥3 studies and are most informative with ≥10 studies).

**Supplementary Text S3. Conversion Rate Considerations**

Conversion from minimally invasive to open surgery is an inherent aspect of laparoscopic emergency colorectal surgery. The included studies reported conversion rates ranging from 4.5% (Vennix 2016) to 33.7% (Le 2024).

**Factors affecting conversion rates:**

Definition: Some studies defined conversion as any open incision; others required completion of the operation open

Era: Earlier studies may have higher rates due to evolving technique

Patient selection: Database studies with broad inclusion may have higher rates

Surgeon experience: High-volume centers report lower conversion rates

Disease severity: Hinchey IV cases have higher conversion rates

Analytical approach: Analyses followed each source study's reported arm definitions. Most studies retained converted cases within the MIS group (intention-to-treat-consistent), although conversion handling was not uniform across studies and could not be fully standardized. This approach:

Reflects real-world effectiveness rather than idealized efficacy

Avoids bias from excluding complex cases

Uses each source study's reported arm definitions consistently for extraction

Interpretation: Higher conversion rates in contemporary database studies (Le 2024: 33.7%) likely reflect:

Appropriate surgical judgment in challenging cases

Inclusive definitions capturing any open assistance

Broad patient populations including severe disease

Not necessarily inferior technique

The observed benefits of MIS despite high conversion rates suggest that the intention to perform MIS and initial laparoscopic exploration may confer advantages even when conversion is ultimately required (e.g., smaller incision, better visualization, less tissue trauma during initial assessment).

**Supplementary Table S9. GRADE Evidence Profile**

**Certainty Assessment**

| **Outcome** | **№ of studies** | **Study design** | **Risk of bias** | **Inconsistency** | **Indirectness** | **Imprecision** | **Other** | **Certainty** | **Importance** |
| --- | --- | --- | --- | --- | --- | --- | --- | --- | --- |
| 30-Day Mortality | 6 | non-randomised | not seriousᵃ | not seriousᵇ | not seriousᶜ | not seriousᵈ | noneᵉ˒ᶠ | ⨁⨁◯◯ LOW | CRITICAL |
| Major Complications (CD ≥III) | 3 | non-randomised | seriousᵍ | not seriousʰ | not seriousⁱ | seriousq | noneʲ | ⨁◯◯◯ VERY LOW | CRITICAL |
| Overall Morbidity (CD I–IV) | 5 | non-randomised | seriousᵏ | seriousˡ | not seriousⁱ | seriousᵐ | noneʲ | ⨁◯◯◯ VERY LOW | CRITICAL |
| Unplanned Reoperation | 6 | non-randomised | not seriousⁿ | not seriousʰ | not seriousⁱ | not seriousᵒ | noneʲ | ⨁⨁◯◯ LOW | CRITICAL |
| Length of Stay (days) | 6 | non-randomised | not seriousⁿ | seriousˡ | not seriousⁱ | not serious | noneʲ | ⨁◯◯◯ VERY LOW | IMPORTANT |
| Operative Time (min) | 6 | non-randomised | not seriousⁿ | seriousˡ | not seriousⁱ | not serious | noneᵈ,ʲ | ⨁◯◯◯ VERY LOW | IMPORTANT |
| Incisional SSI | 5 | non-randomised | not seriousⁿ | seriousˡ | not seriousⁱ | not serious | noneʲ˒ᵖ | ⨁◯◯◯ VERY LOW | IMPORTANT |
| Organ-Space SSI | 4 | non-randomised | not seriousⁿ | not serious | not seriousⁱ | seriousᵐ | noneʲ | ⨁◯◯◯ VERY LOW | IMPORTANT |

**Summary of Findings**

| **Outcome** | **MIS Events/Total** | **Open Events/Total** | **Relative Effect (95% CI)** | **Absolute Effect (95% CI)** |
| --- | --- | --- | --- | --- |
| 30-Day Mortality | 82/1,821 (4.5%) | 683/7,816 (8.7%) | RR 0.48 (0.40–0.59) | 45 fewer per 1,000 (52 fewer to 36 fewer) |
| Major Complications (CD ≥III) | 21/138 (15.2%) | 32/181 (17.7%) | RR 0.63 (0.36–1.08) | 66 fewer per 1,000 (113 fewer to 14 more) |
| Overall Morbidity (CD I-IV) | 98/241 (40.7%) | 144/272 (52.9%) | RR 0.70 (0.47–1.03) | 159 fewer per 1,000 (281 fewer to 16 more) |
| Unplanned Reoperation | 144/1,821 (7.9%) | 623/7,816 (8.0%) | RR 0.98 (0.78–1.22) | 2 fewer per 1,000 (18 fewer to 18 more) |
| Length of Stay (days) | n=1,821 | n=7,816 | — | MD 2.55 days lower (4.37 to 0.74 lower) |
| Operative Time (min) | n=1,821 | n=7,816 | — | MD 18.27 min higher (0.89 higher to 35.64 higher) |
| Incisional SSI | 76/1,761 (4.3%) | 576/7,799 (7.4%) | RR 0.30 (0.12–0.75) | 52 fewer per 1,000 (65 fewer to 18 fewer) |
| Organ-Space SSI | 45/1,722 (2.6%) | 139/7,721 (1.8%) | RR 1.22 (0.47–3.17) | 4 more per 1,000 (10 fewer to 39 more) |

**Explanations**

ᵃ Risk of bias not downgraded. Over 87% of statistical weight derives from studies with moderate risk of bias: Le 2024 (86.9% weight) used entropy balancing with 13 covariates achieving standardized mean differences <0.10 for all variables; Turley 2013 (0.6%) and Vennix 2016 (0.4%) used propensity score matching with good covariate balance. Studies with serious risk of bias (Paasch 2022, Letarte 2015, Cassini 2017) contribute only 2.1% of total weight, insufficient to materially affect the pooled estimate.

ᵇ No downgrade for inconsistency. I²=0% indicates absence of statistical heterogeneity (Q-statistic P=0.60). Five of the six studies showed point estimates favoring minimally invasive surgery, with individual risk ratios ranging from 0.24 (Letarte 2015) to 1.00 (Cassini 2017, exactly at the null); the consistent direction and absence of statistical heterogeneity support no downgrade.

ᶜ No downgrade for indirectness. All studies directly compare emergency minimally invasive versus open colectomy for complicated diverticulitis (Hinchey II–IV) in emergency settings. Population characteristics (mean age 58–67 years, ASA II–III predominant), intervention (laparoscopic or robotic-assisted colectomy), comparator (open colectomy), and outcome (30-day all-cause mortality) align precisely with the review question. No surrogate outcomes used.

ᵈ No downgrade for imprecision. Total 765 mortality events across 9,637 patients exceeds optimal information size threshold (approximately 300–400 events for 25% relative risk reduction). The 95% confidence interval (0.40–0.59) is narrow, does not cross the null value (RR=1.0), and excludes clinically insignificant effects. Sample size provides >95% power to detect the observed effect size.

ᵉ No downgrade for publication bias. Funnel plot (Supplementary Figure S2) shows no obvious asymmetry on visual inspection. Egger's test (P=0.44) and Begg's test (P=0.85) showed no statistical evidence of publication bias. The generally consistent direction of effect and inclusion of both large database studies (Le 2024, Turley 2013) and smaller institutional series suggest reasonably representative literature. However, power to detect bias is limited with only 6 studies.

ᶠ Not upgraded for large effect. Although the pooled risk ratio (0.48) meets the GRADE threshold (RR <0.5), the estimate derives 86.9% of its statistical weight from a single database study (Le 2024) and is no longer statistically significant on leave-one-out excluding that study (RR 0.61, 95% CI 0.35–1.06). Certainty was therefore rated Low rather than upgraded to Moderate.

ᵍ Downgraded one level for risk of bias. For this outcome, 66% of statistical weight derives from studies with serious risk of bias: Paasch 2022 (46.1% weight) adjusted for only 3 variables without propensity matching; Letarte 2015 (20.0% weight) had no statistical adjustment. Only Vennix 2016 (33.9% weight) used propensity score matching with moderate risk.

ʰ No downgrade for inconsistency. I²=0% indicates no statistical heterogeneity. Point estimates show consistent direction across studies.

ⁱ No downgrade for indirectness. All studies directly compare emergency minimally invasive versus open colectomy for complicated diverticulitis (Hinchey II–IV). Population, intervention, comparator, and outcomes align with the review question.

ʲ No downgrade for publication bias. Funnel plot analysis not feasible due to small number of studies. No evidence of selective reporting; comprehensive search included grey literature and trial registries.

ᵏ Downgraded one level for risk of bias. For this outcome, 59% of statistical weight derives from studies with serious risk of bias: Paasch 2022 (26.3%), Cassini 2017 (17.1%), and Letarte 2015 (16.0%) lacked propensity matching or comprehensive covariate adjustment. Only Vennix 2016 (23.9%) and Turley 2013 (16.7%) used propensity score matching.

ˡ Downgraded one level for inconsistency due to substantial statistical heterogeneity, likely reflecting varying outcome definitions, healthcare settings, and patient populations across studies.

ᵐ Downgraded one level for imprecision. The 95% confidence interval crosses the null value and includes both clinically important benefit and no effect (or harm). The result is not statistically significant.

ⁿ Risk of bias not downgraded. Using weight-based assessment per GRADE guidance, majority of statistical weight (>85%) derives from studies with moderate risk of bias employing rigorous confounding control: Le 2024 used entropy balancing with 13 covariates; Turley 2013 and Vennix 2016 used propensity score matching. Studies with serious risk of bias (Paasch 2022, Letarte 2015, Cassini 2017) contribute minimal weight to pooled estimates.

ᵒ Imprecision not downgraded despite confidence interval crossing null. This represents a precise estimate of no clinically meaningful difference between groups with adequate total events. The narrow confidence interval excludes large beneficial or harmful effects.

ᵖ No upgrade for large effect despite RR meeting threshold (<0.5). Per GRADE guidance, upgrade for large effect requires consistent evidence. Substantial heterogeneity (I²>50%) indicates inconsistent effect sizes across studies, failing the consistency requirement for large effect upgrade.

q Major complications: the unmodified HKSJ interval (0.55 to 0.72) is anti-conservative with three studies and I²=0%; the conventional DerSimonian-Laird (Wald-type) random-effects 95% CI (0.36 to 1.08) is reported, crosses the null, and is not statistically significant.

**GRADE Working Group Grades of Evidence**

⨁⨁⨁⨁ HIGH: Very confident that the true effect lies close to the estimate of effect

⨁⨁⨁◯ MODERATE: Moderately confident in the effect estimate; true effect likely close to estimate but may be substantially different

⨁⨁◯◯ LOW: Limited confidence in the effect estimate; true effect may be substantially different

⨁◯◯◯ VERY LOW: Very little confidence in the effect estimate; true effect likely substantially different

**Overall Summary**

| **Certainty Level** | **Outcomes** |
| --- | --- |
| ⨁⨁◯◯ LOW | 30-Day Mortality, Unplanned Reoperation |
| ⨁◯◯◯ VERY LOW | Major Complications, Overall Morbidity, Length of Stay, Operative Time, Incisional SSI, Organ-Space SSI |

Interpretation: Low certainty evidence is consistent with a mortality reduction with minimally invasive surgery, although the pooled estimate depends heavily on one database study and is no longer statistically significant when that study is excluded. Low certainty for reoperation indicates no difference between approaches. Very low certainty for other outcomes reflects serious risk of bias in weight-contributing studies, substantial heterogeneity, or statistical imprecision rather than absence of effect.

**SUPPLEMENTARY FIGURES**

**FIGURE S1: Forest Plot - 30-Day Mortality Sensitivity Analysis (Excluding Le 2024)**

***Supplementary Figure S1.*** *Forest plot of 30-day mortality sensitivity analysis excluding Le 2024. Five studies (513 patients) yielded RR 0.61 (95% CI 0.35–1.06; P=0.07; I²=0%). Point estimate maintains directional consistency without achieving statistical significance, demonstrating the influence of Le 2024 on pooled conclusions.*

**FIGURE S2: Funnel Plot - Publication Bias Assessment**

***Supplementary Figure S2.*** *Funnel plot for publication bias assessment of 30-day mortality. Standard error plotted against log risk ratio for all six studies. No obvious asymmetry observed, though power to detect asymmetry is limited with only six studies. Le 2024 positioned at apex (largest, most precise study).*

**FIGURE S3: Forest Plot - Any Stoma at Index Operation**

***Supplementary Figure S3.*** *Forest plot of any stoma at index operation. Four studies showed extreme heterogeneity (I²=98%) precluding meaningful interpretation: RR 0.61 (95% CI 0.27–1.39; P=0.15). Stoma formation reflects institutional protocols and disease severity rather than surgical approach.*

**FIGURE S4: Forest Plot - Protective Diverting Stoma Among Primary Anastomosis Patients**

***Supplementary Figure S4.*** *Forest plot of protective diverting stoma among primary anastomosis patients. Three studies (168 patients) showed no significant difference: RR 0.63 (95% CI 0.07–5.46; P=0.45; I²=72%). Substantial heterogeneity reflects variable surgeon thresholds for diversion.*

**FIGURE S5: Forest Plot - Anastomotic Leak**

***Supplementary Figure S5.*** *Forest plot of anastomotic leak. Two studies (165 patients) with 5 total leaks demonstrated extreme imprecision: RR 2.90 (95% CI 0.01–839.36; P=0.25). Denominator bases differed (Letarte whole-arm; Vennix primary-anastomosis only). Insufficient data preclude meaningful conclusions about anastomotic safety.*
