# Supplementary material for: Emergency minimally invasive versus open left-sided colonic resection for complicated diverticulitis: a systematic review and meta-analysis
Source: BMC Surg. 2026 Jul 21;26:695. doi: 10.1186/s12893-026-04043-3 (PMC13673721; doi:10.1186/s12893-026-04043-3)
Supplement: Supplementary file 2 [file 12893_2026_4043_MOESM2_ESM.docx]

**PRISMA 2020 Checklist**

**Systematic Review: Emergency Minimally Invasive versus Open Left-Sided Colonic Resection for Complicated Diverticulitis**

**PROSPERO Registration:** CRD420251142546

**Main Checklist**

| **Section/Topic** | **#** | **Checklist Item** | **Location Where Item Is Reported** | **Page** |
| --- | --- | --- | --- | --- |
| **TITLE** |  |  |  |  |
| Title | 1 | Identify the report as a systematic review. | Title page | 1 |
| **ABSTRACT** |  |  |  |  |
| Abstract | 2 | See the PRISMA 2020 for Abstracts checklist. | Abstract | 2 |
| **INTRODUCTION** |  |  |  |  |
| Rationale | 3 | Describe the rationale for the review in the context of existing knowledge. | Introduction, paragraphs 1–4 | 3–4 |
| Objectives | 4 | Provide an explicit statement of the objective(s) or question(s) the review addresses. | Introduction, final paragraph | 4 |
| **METHODS** |  |  |  |  |
| Eligibility criteria | 5 | Specify the inclusion and exclusion criteria for the review and how studies were grouped for the syntheses. | Methods: Eligibility Criteria; Supplementary Text S1 | 4; Supp |
| Information sources | 6 | Specify all databases, registers, websites, organisations, reference lists and other sources searched or consulted to identify studies. Specify the date when each source was last searched or consulted. | Methods: Information Sources and Search Strategy | 4–5 |
| Search strategy | 7 | Present the full search strategies for all databases, registers and websites, including any filters and limits used. | Supplementary Table S1 | Supp |
| Selection process | 8 | Specify the methods used to decide whether a study met the inclusion criteria of the review, including how many reviewers screened each record and each report retrieved, whether they worked independently, and if applicable, details of automation tools used in the process. | Methods: Study Selection and Data Extraction; Supplementary Table S7 | 5; Supp |
| Data collection process | 9 | Specify the methods used to collect data from reports, including how many reviewers collected data from each report, whether they worked independently, any processes for obtaining or confirming data from study investigators, and if applicable, details of automation tools used in the process. | Methods: Study Selection and Data Extraction | 5 |
| Data items | 10a | List and define all outcomes for which data were sought. Specify whether all results that were compatible with each outcome domain in each study were sought (e.g. for all measures, time points, analyses), and if not, the methods used to decide which results to collect. | Methods: Outcomes paragraph; Supplementary Table S5 | 5; Supp |
|  | 10b | List and define all other variables for which data were sought (e.g. participant and intervention characteristics, funding sources). Describe any assumptions made about any missing or unclear information. | Methods: Study Selection and Data Extraction; Supplementary Table S6 | 5; Supp |
| Study risk of bias assessment | 11 | Specify the methods used to assess risk of bias in the included studies, including details of the tool(s) used, how many reviewers assessed each study and whether they worked independently, and if applicable, details of automation tools used in the process. | Methods: Risk of Bias Assessment; Supplementary Table S3 (outcome-level ROBINS-I) | 5–6; Supp |
| Effect measures | 12 | Specify for each outcome the effect measure(s) (e.g. risk ratio, mean difference) used in the synthesis or presentation of results. | Methods: Statistical Analysis; Supplementary Text S2 | 6 |
| Synthesis methods | 13a | Describe the processes used to decide which studies were eligible for each synthesis (e.g. tabulating the study intervention characteristics and comparing against the planned groups for each synthesis (item #5)). | Methods: Statistical Analysis | 6 |
|  | 13b | Describe any methods required to prepare the data for presentation or synthesis, such as handling of missing summary statistics, or data conversions. | Methods: Statistical Analysis; Supplementary Table S6 (median-to-mean conversions) | 6; Supp |
|  | 13c | Describe any methods used to tabulate or visually display results of individual studies and syntheses. | Methods: Statistical Analysis; Figures 2–9 | 6 |
|  | 13d | Describe any methods used to synthesize results and provide a rationale for the choice(s). If meta-analysis was performed, describe the model(s), method(s) to identify the presence and extent of statistical heterogeneity, and software package(s) used. | Methods: Statistical Analysis; Supplementary Text S2 | 6; Supp |
|  | 13e | Describe any methods used to explore possible causes of heterogeneity among study results (e.g. subgroup analysis, meta-regression). | Methods: Statistical Analysis; Supplementary Table S4 | 6; Supp |
|  | 13f | Describe any sensitivity analyses conducted to assess robustness of the synthesized results. | Methods: Statistical Analysis; Supplementary Table S4; Supplementary Figure S1 | 6; Supp |
| Reporting bias assessment | 14 | Describe any methods used to assess risk of bias due to missing results in a synthesis (arising from reporting biases). | Methods: Statistical Analysis; Supplementary Table S8 | 6; Supp |
| Certainty assessment | 15 | Describe any methods used to assess certainty (or confidence) in the body of evidence for an outcome. | Methods: Certainty of Evidence; Table 3 (GRADE Summary of Findings) | 7 |
| **RESULTS** |  |  |  |  |
| Study selection | 16a | Describe the results of the search and selection process, from the number of records identified in the search to the number of studies included in the review, ideally using a flow diagram. | Results: Study Selection; Figure 1 (PRISMA flow diagram); Supplementary Table S2 | 7–8 |
|  | 16b | Cite studies that might appear to meet the inclusion criteria, but which were excluded, and explain why they were excluded. | Supplementary Table S2 (106 excluded studies with reasons) | Supp |
| Study characteristics | 17 | Cite each included study and present its characteristics. | Results: Study Characteristics; Table 1; Table 2 | 9–10 |
| Risk of bias in studies | 18 | Present assessments of risk of bias for each included study. | Results: Risk of Bias Assessment; Supplementary Table S3 (outcome-level ROBINS-I) | 11; Supp |
| Results of individual studies | 19 | For all outcomes, present, for each study: (a) summary statistics for each group (where appropriate) and (b) an effect estimate and its precision (e.g. confidence/credible interval), ideally using structured tables or plots. | Results: Primary and Secondary Outcomes; Figures 2–9; Table 2 | 11–20 |
| Results of syntheses | 20a | For each synthesis, briefly summarise the characteristics and risk of bias among contributing studies. | Results: sections for each outcome | 11–20 |
|  | 20b | Present results of all statistical syntheses conducted. If meta-analysis was done, present for each the summary estimate and its precision (e.g. confidence/credible interval) and measures of statistical heterogeneity. If comparing groups, describe the direction of the effect. | Results: Primary and Secondary Outcomes; Figures 2–9; Table 2 | 11–20 |
|  | 20c | Present results of all investigations of possible causes of heterogeneity among study results. | Results: Sensitivity Analyses; Supplementary Table S4 | 12; Supp |
|  | 20d | Present results of all sensitivity analyses conducted to assess the robustness of the synthesized results. | Results: Sensitivity Analyses; Supplementary Table S4; Supplementary Figure S1 | 12; Supp |
| Reporting biases | 21 | Present assessments of risk of bias due to missing results (arising from reporting biases) for each synthesis assessed. | Results: Publication Bias; Supplementary Table S8; Supplementary Figure S2 | 17; Supp |
| Certainty of evidence | 22 | Present assessments of certainty (or confidence) in the body of evidence for each outcome assessed. | Results: Certainty of Evidence; Table 3 (GRADE Summary of Findings using GRADEpro GDT) | 18 |
| **DISCUSSION** |  |  |  |  |
| Discussion | 23a | Provide a general interpretation of the results in the context of other evidence. | Discussion, paragraphs 1–4 | 21–23 |
|  | 23b | Discuss any limitations of the evidence included in the review. | Discussion: Limitations paragraph | 23 |
|  | 23c | Discuss any limitations of the review processes used. | Discussion: Limitations paragraph | 23 |
|  | 23d | Discuss implications of the results for practice, policy, and future research. | Discussion: Clinical Implications; Future Research Directions | 23–24 |
| **OTHER INFORMATION** |  |  |  |  |
| Registration and protocol | 24a | Provide registration information for the review, including register name and registration number, or state that the review was not registered. | Methods: Protocol and Registration; Abstract | 4; 2 |
|  | 24b | Indicate where the review protocol can be accessed, or state that a protocol was not prepared. | Methods: Protocol and Registration (PROSPERO CRD420251142546) | 4 |
|  | 24c | Describe and explain any amendments to information provided at registration or in the protocol. | Methods: Protocol and Registration (protocol version 5.2; no post hoc changes) | 4 |
| Support | 25 | Describe sources of financial or non-financial support for the review, and the role of the funders or sponsors in the review. | Funding Statement | 25 |
| Competing interests | 26 | Declare any competing interests of review authors. | Conflicts of Interest Statement | 25 |
| Availability of data, code and other materials | 27 | Report which of the following are publicly available and where they can be found: template data collection forms; data extracted from included studies; data used for all analyses; analytic code; any other materials used in the review. | Data Availability Statement; Supplementary Materials | 25; Supp |

**PRISMA 2020 for Abstracts Checklist**

| **Section** | **Item** | **Checklist Item** | **Reported** |
| --- | --- | --- | --- |
| **Title** | 1 | Identify the report as a systematic review. | ✓ |
| **Background** | 2 | Provide an explicit statement of the main objective(s) or question(s) the review addresses. | ✓ |
| **Methods** | 3 | Specify the inclusion and exclusion criteria for the review. | ✓ |
|  | 4 | Specify the information sources (e.g. databases, registers) used to identify studies and the date when each was last searched. | ✓ |
|  | 5 | Specify the methods used to assess risk of bias in the included studies. | ✓ |
|  | 6 | Specify the methods used to present and synthesise results. | ✓ |
| **Results** | 7 | Give the total number of included studies and participants and summarise relevant characteristics of studies. | ✓ |
|  | 8 | Present results for main outcomes, preferably indicating the number of included studies and participants for each. If meta-analysis was done, report the summary estimate and confidence/credible interval. If comparing groups, indicate the direction of the effect (i.e. which group is favoured). | ✓ |
| **Discussion** | 9 | Provide a brief summary of the limitations of the evidence included in the review (e.g. study risk of bias, inconsistency and imprecision). | ✓ |
|  | 10 | Provide a general interpretation of the results and important implications. | ✓ |
| **Other** | 11 | Specify the primary source of funding for the review. | ✓ |
|  | 12 | Provide the register name and registration number. | ✓ |

**Supplementary Materials Structure**

**Supplementary Tables**

| **Table** | **Content** |
| --- | --- |
| S1 | Complete search strategy for all databases |
| S2 | List of excluded studies with reasons (n=106) |
| S3 | Detailed ROBINS-I assessment at **outcome level** |
| S4 | Sensitivity and subgroup analyses (including leave-one-out) |
| S5 | Outcome definition harmonization |
| S6 | Median-to-mean conversion details |
| S7 | Inter-rater reliability for study selection (κ=0.92, κ=0.88) |
| S8 | Publication bias assessment |
| S9 | GRADE evidence profile with detailed explanations |

**Supplementary Figures**

| **Figure** | **Content** |
| --- | --- |
| S1 | Sensitivity analysis excluding Le 2024 (influence plot) |
| S2 | Funnel plot for publication bias |
| S3 | Forest plot: Any stoma at index operation (I²=98%, extreme heterogeneity) |
| S4 | Forest plot: Protective diverting stoma among primary anastomosis |
| S5 | Forest plot: Anastomotic leak (insufficient data) |

**Supplementary Texts**

| **Text** | **Content** |
| --- | --- |
| S1 | Definition of "emergency surgery" |
| S2 | Statistical methods detail (including justification for pooling adjusted/unadjusted data) |
| S3 | Conversion rate considerations (ITT analysis implications) |

**Main Manuscript Tables and Figures**

**Tables**

| **Table** | **Content** | **Location** |
| --- | --- | --- |
| 1 | Characteristics of included studies | Results |
| 2 | Baseline patient characteristics | Results |
| 3 | GRADE Summary of Findings (GRADEpro GDT) | Results |

**Figures**

| **Figure** | **Content** |
| --- | --- |
| 1 | PRISMA 2020 flow diagram |
| 2 | Forest plot: 30-day mortality (primary outcome) |
| 3 | Forest plot: Major complications CD ≥III (primary outcome) |
| 4 | Forest plot: Overall morbidity |
| 5 | Forest plot: Unplanned reoperation |
| 6 | Forest plot: Length of hospital stay (scale ±15 days) |
| 7 | Forest plot: Operative time |
| 8 | Forest plot: Incisional surgical site infection |

**Reference**

Page MJ, McKenzie JE, Bossuyt PM, et al. The PRISMA 2020 statement: an updated guideline for reporting systematic reviews. BMJ. 2021;372:n71. doi:10.1136/bmj.n71
